# Cadmium tolerance and taxonomic diversity of cultivable bacteria isolated from *Inga* sp. nodules, *Quararibea* sp. roots and rhizosphere soil in cocoa agroforestry systems of the Peruvian Amazon

**DOI:** 10.3389/fmicb.2026.1845000

**Published:** 2026-08-07

**Authors:** Jessenia Shirley Ramos Armaulia, Santos Triunfo Leiva Espinoza, Segundo Manuel Oliva Cruz

**Affiliations:** Instituto de Investigación para el Desarrollo Sustentable de Ceja de Selva (INDES-CES), Universidad Nacional Toribio Rodríguez de Mendoza de Amazonas, Chachapoyas, Amazonas, Peru

**Keywords:** bacterial tolerance, bioremediation, cadmium, hormesis, *Inga*, non-rhizobial endophytes, Peruvian Amazon, *Quararibea*

## Abstract

Cadmium (Cd) contamination in the Peruvian Amazon cocoa soils threatens production sustainability and international market access. This study characterized the taxonomic and genomic diversity of bacteria isolated from *Inga* sp. nodules, *Quararibea* sp. roots, and rhizosphere soil in cocoa agroforestry systems in Bagua, Amazonas, Peru (1.02–3.54 mg Cd kg^−1^), and evaluated the Cd tolerance phenotypes *in vitro*. From 93 initial isolates, 53 grew under cadmium screening; 23 were selected for Sanger sequencing, yielding 12 high-quality 16S rRNA sequences suitable for downstream phylogenetic analysis (GenBank: PZ012327–PZ012338). These strains were identified via 16S rRNA gene sequencing and BOX-PCR fingerprinting, revealing distribution across three bacterial phyla (Proteobacteria, Firmicutes, Actinobacteria) and five classes. Notably, no rhizobia sensu stricto were recovered from *Inga* sp. nodules; all nodule-derived isolates were non-rhizobial endophytes (NREs) belonging to *Achromobacter*, *Pseudomonas*, *Klebsiella*, *Priestia*, and *Glutamicibacter*. BOX-PCR identified 12 unique genotypes (Shannon H′ = 2.485) distributed in four genomic clusters unrelated to phylogenetic affiliation. The Cd tolerance (25–200 ppm) was assessed via OD₆₀₀ in TY medium. Tolerance indices (0.190–0.643) classified strains into quartiles: highly tolerant (*n* = 3), tolerant (*n* = 3), sensitive (*n* = 3), and highly sensitive (*n* = 3). JSRA_S05 (*K. va*r*iicola*) showed clear hormesis (TI = 1.235 at 25 ppm), whereas JSRA_S08 (*Glutamicibacter* sp.) exhibited a possible hormetic tendency (TI = 1.012 at 50 ppm). IC₅₀ values (0.18–719.7 ppm) showed low correlation with TI (*ρ* < 0.1), indicating complementary dimensions of tolerance. Tolerance was strain-specific, as evidenced by opposite responses of two *K. variicola* strains (highly tolerant JSRA_S05 versus highly sensitive JSRA_N14). Strains associated with *Quararibea* sp. were consistently among the most tolerant. Three strains (JSRA_S05, JSRA_S08, and JSRA_R15), combining high physiological tolerance with taxonomic and source diversity, are proposed as priority candidates for functional evaluation in biosorption, bioaccumulation, and plant-inoculation assays. These culture-dependent findings indicate that, under the cultivation conditions employed, the cultivable bacterial fraction recovered from Cd-impacted Inga sp. nodules in Amazonian cocoa agroecosystems is dominated by non-rhizobial endophytes, representing a promising biological resource for further bioremediation studies. Whether classical rhizobia are truly absent or simply not retrieved under these conditions requires culture-independent confirmation.

## 1 Introduction

Heavy metal pollution poses a serious challenge to human health and the integrity of global ecosystems (Rajaganapa et al., 2011). Cadmium (Cd) occurs naturally in soils as a result of geogenic processes such as volcanic eruptions, weathering of parent rocks, and pedogenic evolution (Cullen and Maldonado, 2013; Gramlich et al., 2018). In addition to these natural sources, anthropogenic activities intensify Cd accumulation in agricultural soils, including mining operations, the manufacture of plastics and pigments, the application of phosphate fertilizers (Siripornadulsil and Siripornadulsil, 2013), and irrigation with untreated wastewater (Kirkham, 2006; Maddela et al., 2020).

In the Peruvian Amazon, cadmium is one of the main limiting factors for the sustainable production of cacao (*Theobroma cacao* L.). Recent studies have shown that Cd concentrations in Peruvian cacao beans exhibit high spatial variability, with averages ranging from 0.076 to 0.99 mg/kg (Thomas et al., 2023). Elevated Cd concentrations are mainly restricted to the northern regions of the country—the departments of Tumbes, Piura, Amazonas, and Loreto as well as in localized hotspots in the central regions of Huánuco and San Martín (Thomas et al., 2023; Vallejos-Torres et al., 2025). This heterogeneous distribution is strongly influenced by geological factors, alluvial deposition, soil pH, seasonal rainfall patterns, and, to a lesser extent, mining activities (Guarín et al., 2023).

Beyond its environmental impact, Cd in cocoa is also a regulatory concern: the European Union maximum limits for Cd in chocolate products (European Union, 2023) threaten access to international markets for South American producers (Thomas et al., 2023). Cd also exerts selective pressure on soil and rhizosphere microbial communities, favoring bacterial lineages with adaptive strategies against metal stress (Rojas Briceño et al., 2021; Vallejos-Torres et al., 2025).

Cocoa agroforestry systems represent complex agricultural models that integrate multipurpose tree species, particularly legumes of the genus Inga and tree species of the genus *Quararibea* (Malvaceae), recognized for their ability to accumulate heavy metals in plant tissues (Castillo et al., 2016). These systems harbor structured and functionally diverse root microbiomes that include rhizosphere bacteria, root endophytes, and nodule-associated microorganisms (Vallejos-Torres et al., 2023). These microbial consortia play key roles in plant nutrition, abiotic stress tolerance, and agroecosystem resilience to environmental disturbances.

The synergistic interaction between plants and microorganisms in agroecosystems contaminated with heavy metals constitutes a fundamental mechanism for biological remediation. Plant-associated microorganisms can modify the bioavailability of heavy metals through various mechanisms, including methylation, alteration of soil pH, redox processes, and secretion of siderophores, organic acids, and biosurfactants (Ma et al., 2016). These microorganisms represent promising biotechnological alternatives for mitigating the impact of Cd in cocoa agroecosystems (Bashir et al., 2024; Feng et al., 2024).

Traditionally, legume nodules have been conceptualized as specialized niches dominated exclusively by nitrogen-fixing rhizobial bacteria of the Rhizobiaceae family. However, recent molecular evidence based on high-throughput sequencing of the 16S rRNA gene has revealed that root nodules harbor remarkably diverse bacterial communities, including abundant non-rhizobial lineages (Hnini and Aurag, 2024). Metagenomic studies have detected between 87 and 99% of the total bacterial population in nodules corresponding to non-rhizobial endophytes (NREs), coexisting with nitrogen-fixing symbionts (Hassen et al., 2025).

These non-rhizobial endophytes include genera such as *Pseudomonas*, *Bacillus*, *Paenibacillus*, *Enterobacter*, *Chryseobacterium*, *Sphingobacterium*, and members of the Enterobacteriaceae family. Although these bacteria do not induce nodule formation on their own, they confer significant benefits to the host through mechanisms that include atmospheric nitrogen fixation, solubilization of inorganic phosphates, production of phytohormones, synthesis of siderophores, and ACC deaminase activity, thereby improving plant growth and resistance to abiotic stresses (Hassen et al., 2025). Particularly under heavy metal stress conditions, it has been documented that the diversity and functionality of non-rhizobial endophytes increase, suggesting an adaptive role in the colonization of metal-rich environments (Safronova et al., 2012).

In tropical leguminous trees, studies on nodular communities remain limited. Dos Santos et al. (2020) and Rollo et al. (2020) have highlighted the potential of *Inga edulis* as a promising species for the phytoremediation of cadmium-contaminated soils, emphasizing its ability to accumulate heavy metals in plant tissues. This phytoremediation capacity does not depend exclusively on the physiological attributes of the host plant, but is closely linked to the endophytic microorganisms inhabiting its nodules and the surrounding rhizosphere. Similarly, species of the genus *Quararibea*, although not legumes, have demonstrated the ability to capture heavy metals and accumulate essential elements such as Fe, Mg, Cu, and Na (Castillo et al., 2016), suggesting that the microbiomes associated with these tree species may harbor bacteria with mechanisms for metal tolerance and mobilization.

Xiang et al. (2023) identified bacterial endophytes in root nodules of *Flemingia falcata,* reporting that Proteobacteria constitute the dominant phylum with relative abundances ranging from 42 to 90% in all analyzed samples, reinforcing the hypothesis that nodules of tropical tree legumes represent reservoirs of functional bacterial diversity beyond classical rhizobia.

Non-rhizobial bacteria of families such as Bacillaceae, Enterobacteriaceae and Pseudomonadaceae are known to display a range of Cd-tolerance mechanisms (efflux pumps, exopolysaccharide-mediated cation sequestration, intracellular bioaccumulation, redox biotransformation and, at low doses, hormetic responses) (Ma et al., 2016; Agathokleous et al., 2022).

Bacteria isolated from soils contaminated with heavy metals tend to exhibit significantly higher tolerance levels than those from uncontaminated soils, indicating processes of natural selection and local adaptation (Goyal and Habtewold, 2023). Recent studies have shown that heavy metal-tolerant bacteria associated with legumes not only improve plant growth under metal stress but also modulate the composition of the rhizosphere microbiome, favoring colonization by other microorganisms with plant growth-promoting capabilities (Etesami and Adl, 2020; Etesami and Santoyo, 2025). In cocoa systems, Feria-Cáceres et al. (2022) demonstrated that native bacteria isolated from Cd-contaminated cocoa soils exhibit metal tolerance and bioaccumulation capabilities, highlighting genera such as *Klebsiella*, *Enterobacter*, *Bacillus*, and *Cupriavidus*, which coincide with lineages frequently reported in the rhizospheres of phytoremediating plants.

However, despite this growing body of knowledge, the ecological distribution, taxonomic diversity, and functional relevance of non-rhizobial bacteria associated with leguminous trees in cadmium-contaminated Amazonian cocoa systems remain unexplored, representing a critical knowledge gap for the development of biotechnological bioremediation strategies.

In Peru, studies on microorganisms associated with cacao have focused predominantly on arbuscular mycorrhizal fungi (Vallejos-Torres et al., 2023) or on classical rhizobia isolated from leguminous herbaceous plants, leaving a significant gap regarding: (i) the taxonomic and genomic diversity of non-rhizobial bacteria in nodules of multipurpose leguminous trees (e.g., *Inga* spp.) in cocoa agroforestry systems; (ii) the functional phenotypes of these bacterial communities in response to cadmium; and (iii) their potential application in plant-assisted bioremediation strategies.

This study is justified by integrating taxonomic analyses via 16S rRNA marker gene sequencing, characterization of genomic diversity via BOX-PCR fingerprinting, and evaluation of *in vitro* cadmium dose–response phenotypes. This multidimensional approach allows for a realistic and robust assessment of the bioremediation potential of native bacteria isolated from contaminated cocoa soils. The identification of indigenous bacterial strains with demonstrated cadmium tolerance represents a critical step toward the development of specific bioinoculants for Amazonian agroforestry systems, contributing to the sustainability of cocoa production in the face of metal contamination challenges.

The objective of this study was to characterize the taxonomic and genomic diversity of bacteria isolated from *Inga* spp. nodules, *Quararibea* spp. roots, and rhizosphere soil in cocoa agroforestry systems in Bagua, Amazonas, Peru, and to evaluate their cadmium tolerance phenotypes in vitro as a criterion for bioremediation potential. It is hypothesized that bacterial isolates associated with phytoremediating plants in Cd-contaminated soils exhibit differential tolerance to the metal, with dose-dependent response profiles reflecting diverse adaptive strategies, including hormetic responses.

## 2 Materials and methods

### 2.1 Study area and agroforestry system

Note on nomenclature. Throughout this paper we use *Inga* sp. and *Quararibea* sp. (singular) to refer to the single morphotype of each genus identified at the sampling sites by morphological and molecular characterization. The plural form “spp.” appears in the text only when referring to these genera in the broader literature or as collective categories.

**Figure 1:**
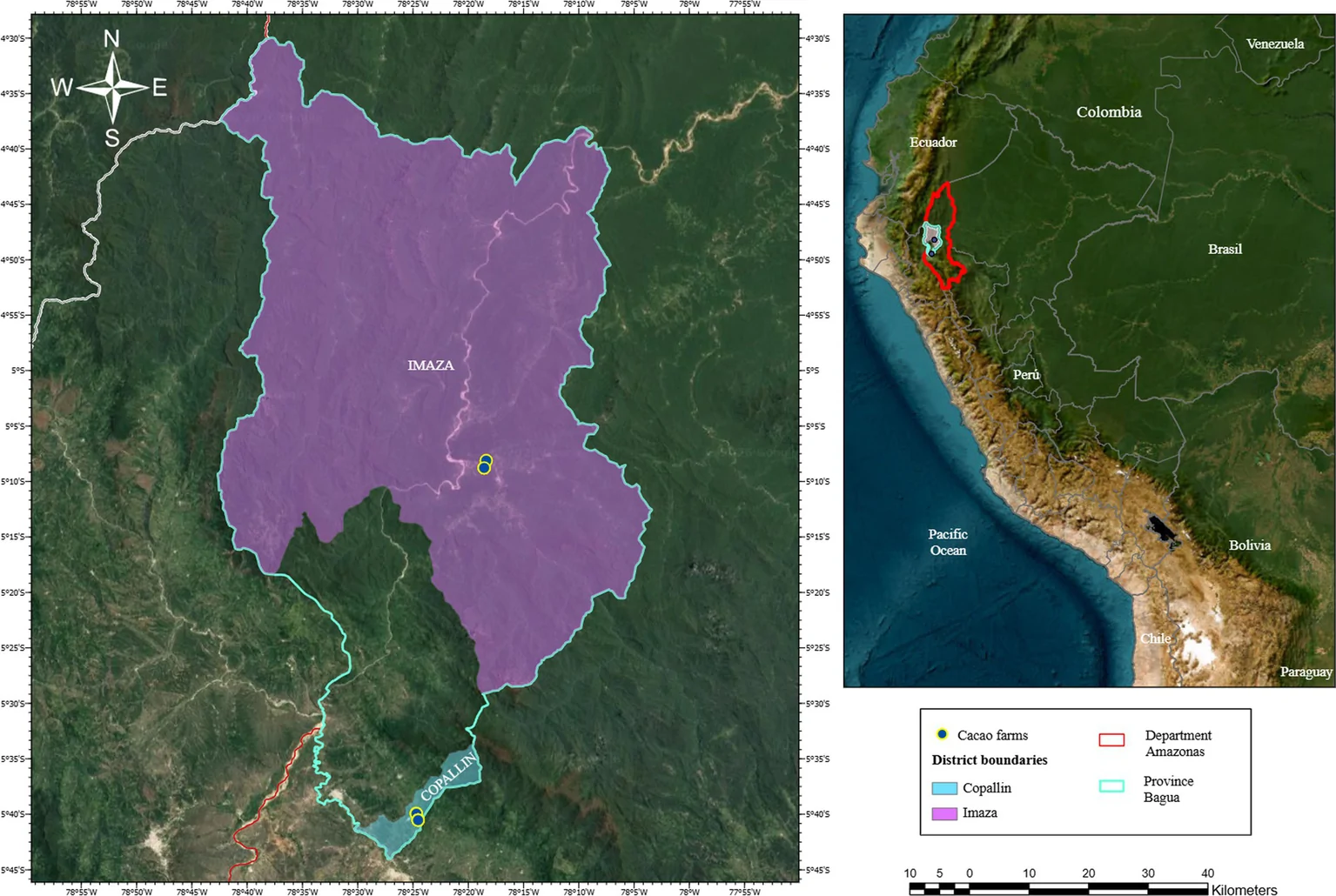
Location of the study area in Bagua Province, Amazonas Region, Peru. Map showing the location of cacao agroforestry farms sampled in the districts of Imaza and Copallín, Bagua Province, Amazonas Region, Peru (approximately 5°38’S, 78°31’W, 400–800 m a.s.l.). Yellow circles indicate the sampled cacao farms associated with *Inga* spp. and *Quararibea* spp. shade trees. The inset map (right) shows the location of the Amazonas department within Peru and South America. Map elaborated using ArcGIS.

The study was conducted in cocoa (*Theobroma cacao* L.) agroforestry systems located in the province of Bagua, Amazonas region, Peru, within the districts of Imaza and Copallín (and their constituent populated centers of Chiriaco, Imaza, Cajaruro, La Lluhuana and Copallín town), at approximately 5°20–5°45′S and 78°15–78°35′W, ranging from 288 to 965 m a.s.l. (Figure 1). All field sampling was carried out in Bagua, whereas laboratory processing (isolation, sequencing and Cd tolerance assays) was conducted at the INDES-CES facility of the Universidad Nacional Toribio Rodríguez de Mendoza de Amazonas, Chachapoyas. The agroforestry systems studied are characterized by the cultivation of cacao under the shade provided by multipurpose tree species, mainly legumes of the genera *Inga* spp. and *Quararibea* spp., recognized for their capacity to accumulate heavy metals (Berto et al., 2015; Aguilar et al., 2023).

The study area was selected for its documented history of Cd contamination. A characterization of soils in the same agroforestry zone, carried out under the FITOCADMIO project, reported soil Cd concentrations ranging from 1.02 to 3.54 mg Cd kg^−1^, with several plots exceeding the limit of 1.4 mg Cd kg^−1^ established by Peruvian environmental regulations for agricultural soils (MINAM, 2017; Oliva et al., 2020). Soil pH ranged from 4.4 to 8.1, organic matter from 1.0 to 6.5%, and textures from sandy loam to clay (Table 1). These values are consistent with previous regional surveys of Peruvian cacao soils (Thomas et al., 2023; Vallejos-Torres et al., 2025). The 36 plots sampled in the present study (see section 2.2) are located within this characterized area; they were not individually re-analyzed for soil Cd, and the available soil characterization of the area is therefore used as the edaphic context of this study. The region’s climate is humid tropical, with an average annual temperature of 24–26 °C and annual precipitation between 1,200–1,800 mm, concentrated between October and May (Oliva et al., 2020). The predominant soils in the Peruvian Amazon belong to the Ultisols, Alfisols, and Inceptisols orders (equivalent to Acrisols, Luvisols/Lixisols, and Cambisols in the WRB classification), derived from sedimentary and alluvial parent materials, with pH ranging from acidic to neutral and variable organic matter content (Quesada et al., 2011; Oliva et al., 2020).

**Table 1:** Range of soil physicochemical properties of the Bagua agroforestry study area, by district (data from a soil characterization carried out under the FITOCADMIO project; 29 plots; values are min–max).

District	*n*	pH (1:1)	O. M. (%)	N (%)	Texture range	Soil Cd (mg kg^−1^)
Imaza	3	4.42–5.48	1.29–6.07	0.06–0.30	Sandy loam–loam	1.10–1.76
Aramango	2	5.92–6.62	4.31–5.60	0.22–0.28	Clay loam	1.97–3.54
La Peca	15	5.00–7.99	1.03–6.47	0.05–0.32	Sandy loam–clay	1.02–1.62
Copallín	9	5.62–8.06	4.73–6.30	0.24–0.32	Sandy loam–clay	1.28–2.64
Total area	29	4.42–8.06	1.03–6.47	0.05–0.32	Sandy loam–clay	1.02–3.54

Values represent min–max across plots within each district. The data come from a soil characterization of the same agroforestry area carried out under the FITOCADMIO project and are independent of the 36 bacterial-sampling plots described in section 2.2; they are used here as edaphic context.

The interaction between host species (cocoa and tree legumes) and edaphoclimatic conditions, combined with cadmium stress, constitutes an appropriate ecological setting for investigating the diversity and functionality of bacteria associated with nodules and the rhizosphere that have the potential to adapt to heavy metals.

### 2.2 Sampling of nodules and rhizosphere soil

#### 2.2.1 Selection and characterization of plots

Plots were selected through targeted sampling, prioritizing those with: (i) documented cadmium contamination in the soil, (ii) the presence of at least two adult individuals of *Inga* spp. and/or *Quararibea* spp. in association with cacao, (iii) logistical accessibility, and (iv) express authorization from the landowners. In each selected plot, geographic coordinates were recorded using GPS.

A total of 36 sampling units were established across the study area, distributed across the populated centers of Chiriaco, Imaza, Cajaruro, Copallín and La Lluhuana (districts of Imaza and Copallín). Three types of samples were collected, depending on the host species and tissue available at each plot: (i) rhizosphere soil, (ii) root tissue (endosphere) of *Quararibea* sp., and (iii) root nodules of *Inga* sp. (the only nodulating legume host in the system; *Quararibea* sp., a non-leguminous Malvaceae, does not form nodules and was sampled only for rhizosphere soil and root endosphere). The compartment of origin is reflected in the strain code prefix: N = nodule, R = root, S = soil. Plot-level metadata, including coordinates, altitude, host species and sample type, are summarized in Supplementary Table S1.

#### 2.2.2 Rhizosphere soil sampling

Rhizosphere soil sampling was conducted following the guidelines of the Soil Sampling Guide of the Peruvian Ministry of the Environment (MINAM, 2014). In each plot, between 5 and 10 individuals of each target plant species (*Inga* spp. and *Quararibea* spp.) were randomly selected. For each individual, rhizosphere soil samples were collected at a depth of 10–20 cm at four cardinal points distributed radially around the main trunk, at a distance of approximately 50 cm from it.

Using a stainless steel auger (2.5 cm diameter) previously sterilized with 70% ethanol (v/v), approximately 100 g of soil was extracted per cardinal point. The subsamples were homogenized manually by quartering to obtain a representative composite sample of approximately 400 g per individual. The collected soil was stored in sterile, airtight polyethylene bags, properly labeled with the sample code, GPS coordinates, date, and host species.

To minimize cross-contamination between samples, the auger and auxiliary tools (shovel, spatula) were thoroughly cleaned between each sampling point by washing with distilled water, rinsing with 70% ethanol, and air-drying. The samples were kept refrigerated (4–8 °C) during transport to the laboratory (maximum 48 h post-collection) and were immediately processed for bacterial isolation or stored at −20 °C for subsequent physicochemical analysis.

#### 2.2.3 Root nodule sampling

Nodule collection was performed following established protocols for tree legumes (Somasegaran and Hoben, 1994). *Inga* spp. trees were selected that had not received recent (≤6 months) applications of nitrogen fertilizers or phytosanitary products, in order to avoid inhibitory effects on nodule populations. Through careful excavation with a shovel and hoe, the primary root system was exposed at a depth of approximately 20–40 cm and a radial distance of 30–50 cm from the trunk.

The nodule-bearing secondary roots were extracted manually, minimizing mechanical damage to the nodular system. The nodules were cut with previously sterilized fine-tipped scissors, keeping a small fragment of root attached (3–5 mm) to facilitate identification of the nodular tissue during processing. The nodules were superficially washed with running water to remove excess adhered soil and placed in sterile Kraft paper envelopes (avoiding plastic materials that promote condensation and fungal growth).

The nodule samples were stored in a portable cooler (4–10 °C) with ice packs during transport to the laboratory. Nodule processing was performed within 24–48 h after collection. For samples requiring temporary storage, the nodules were partially dehydrated at room temperature (25 °C, 48 h) and stored in paper envelopes at 4 °C for a maximum of 7 days, following the recommendations of Somasegaran and Hoben (1994).

#### 2.2.4 Sampling of *Quararibea* spp. roots for isolation of endophytic bacteria

Since *Quararibea* spp. (family Malvaceae) is not a legume and therefore does not form symbiotic nodules, the sampling focused on collecting secondary roots and rootlets for the isolation of endophytic bacteria from the rhizosphere and internal root tissue. Protocols adapted from studies of endophytic bacteria in non-leguminous tree species (Hardoim et al., 2015; Sahu et al., 2022) were applied.

Apparently healthy adult trees of *Quararibea* spp. were selected, with no visible symptoms of foliar or root disease. Through controlled excavation with a shovel, the root system was exposed to a depth of 15–30 cm and a radial distance of 40–60 cm from the main trunk. Active secondary roots (diameter 2–5 mm) with attached fine rootlets were collected by cutting them with sterile pruning shears into sections approximately 10–15 cm in length.

The roots were immediately placed in sterile, airtight polyethylene bags, with residual moisture maintained by adding sterile moist absorbent paper to prevent tissue desiccation. Excessive washing of the roots in the field was avoided to preserve the rhizosphere and rhizoplane microbiota. The samples were transported in a cooler (4–8 °C) and processed within 24 h of collection.

### 2.3 Bacterial isolation and purification

#### 2.3.1 Isolation of bacteria from root nodules

##### 2.3.1.1 Preparation and surface sterilization of nodules

The collected nodules were processed following modified protocols from Dilworth (2016). Dehydrated nodules were rehydrated by immersion in sterile deionized water for 3–4 h at room temperature. Fresh nodules with attached root tissue were processed directly after preliminary washing with running water to remove soil residues.

Surface sterilization was performed by sequential immersion in: (i) 70% (v/v) ethanol for 60 s, (ii) 3–4% (v/v) with 0.01% Tween 80 for 3–4 min, with gentle vortex agitation at 30-s intervals. The exposure time to NaClO was empirically adjusted based on the size and thickness of the nodular cortex, following the criteria of. Subsequently, the nodules were thoroughly rinsed through six successive washes with sterile distilled water (100 mL per wash, 1 min each) to completely remove disinfectant residues.

The effectiveness of surface sterilization was verified by plating treated nodules onto YMA (Yeast Mannitol Agar) plates, followed by incubation at 28 °C for 5–7 days. The absence of bacterial growth was interpreted as successful sterilization.

##### 2.3.1.2 Extraction and inoculation of nodular endophytic bacteria

The superficially sterilized nodules were processed under aseptic conditions in a laminar flow hood (Labconco). For individual nodules, direct maceration was used: the nodule was compressed with sterile blunt-tipped forceps directly onto the surface of supplemented YMA medium, distributing the bacterial exudate via dilution streaks following a four-quadrant pattern. For larger nodules (>5 mm diameter), maceration in a drop of sterile water was used: the nodule was macerated in 50 μL of 0.85% sterile saline solution (NaCl) on the inverted lid of a Petri dish. Subsequently, 10 μL of the macerate was transferred to YMA plates and spread using a calibrated loop.

The inoculated plates were incubated at 28 ± 2 °C in the dark, with visual monitoring every 24 h for 5–10 days. Bacterial growth was documented photographically, and the time of colony appearance, colony morphology (size, shape, color, transparency, exopolysaccharide production), and Congo Red dye absorption pattern were recorded.

##### 2.3.1.3 Extraction and inoculation of endophytic bacteria

In the laboratory, the roots underwent a sequential separation of microbial compartments following the rhizosphere-rhizoplane-endosphere scheme. First, the adhering rhizosphere soil was removed by vigorous manual shaking. Subsequently, the roots were washed with sterile distilled water to remove residual soil. For the isolation of strict endophytic bacteria from the internal root tissue, the cleaned roots were superficially sterilized by sequential immersion in: (i) 70% (v/v) ethanol for 1–2 min, (ii) 2.5–3.0% (v/v) sodium hypochlorite (NaClO) for 3–5 min, and (iii) five rinses with sterile distilled water (1 min each) (Hardoim et al., 2015; Sahu et al., 2022).

The efficacy of surface sterilization was verified using the imprint method: segments of surface-sterilized roots were pressed onto plates containing YMA medium for 10 s, followed by incubation at 28 °C for 5–7 days. The absence of bacterial growth confirmed the elimination of the epiphytic microbiota. After verification, the surface-sterilized roots were aseptically cut into 0.5–1.0 cm fragments and macerated in sterile 0.85% NaCl saline solution to release endophytic bacteria from the internal tissue, and were immediately processed for bacterial isolation.

##### 2.3.1.4 Isolation of rhizosphere soil bacteria

To isolate cultivable rhizosphere bacteria, the plate serial dilution method was used (Somasegaran and Hoben, 1994). 1.0 g of rhizosphere soil (fresh weight) was weighed aseptically and resuspended in 9 mL of sterile 0.85% saline solution (NaCl) in 15 mL Falcon tubes. The suspension was homogenized by vigorous vortexing (3 min) to detach bacteria adhering to soil particles, following recommendations from Sessitsch et al. (2001).

Starting from the stock suspension (10^−1^), serial decimal dilutions were prepared down to 10^−4^ by successive transfers of 1 mL into 9 mL of sterile saline solution. From the 10^−3^ and 10^−4^ dilutions, aliquots of 100 μL were inoculated onto Petri dishes containing supplemented YMA medium. The inoculum was distributed uniformly over the surface of the agar using a calibrated ring inoculator, employing the plate streaking technique.

The plates were incubated at 28 ± 2 °C in the dark for 5–7 days. Colonies with morphology consistent with rhizobia and non-rhizobial bacteria (circular, convex, mucoid, translucent to creamy-white colonies, with or without Congo Red uptake) were selected for purification through successive subcultures in YMA medium until pure cultures were obtained, verified by microscopic observation and uniform colonial morphology.

##### 2.3.1.5 Preparation of selective culture media

Yeast Mannitol Agar (YMA) was used as the basal medium for the isolation and maintenance of strains, following the formulation by Vincent (1970) and Somasegaran and Hoben (1994): mannitol 10.0 g L^−1^, yeast extract 1.0 g L^−1^, K₂HPO₄ 0.5 g L^−1^, MgSO₄·7H₂O 0.2 g L^−1^, NaCl 0.1 g L^−1^, bacteriological agar 15.0 g L^−1^, pH adjusted to 6.8–7.0 with 1 M HCl or 1 M NaOH.

For presumptive selection of rhizobia, the YMA medium was supplemented with Congo Red 0.0025% (w/v) and Crystal Violet 0.001% (w/v), prepared as filter-sterilized (0.45 μm) stock solutions and added to the medium after autoclaving and cooling to 50–55 °*C. Congo* Red acts as a differential indicator: typical rhizobia do not absorb the dye (white/translucent colonies), whereas most contaminants produce red colonies. Crystal Violet selectively inhibits the growth of fast-growing Gram-positive bacteria (Somasegaran and Hoben, 1994).

The medium was sterilized in an autoclave at 121 °C, 15 psi for 20 min, aseptically distributed into sterile Petri dishes (approximately 20 mL per dish), and solidified at room temperature under aseptic conditions. The plates were stored upside down at 4 °C in sealed plastic bags for up to 4 weeks.

##### 2.3.1.6 Purification and presumptive identification of isolates

Bacterial colonies with colony morphology of interest (mucoid, translucent, slow-to-moderate growth, no Congo Red uptake for presumed rhizobia; or contrasting morphologies for presumed non-rhizobial bacteria) were streaked out on fresh YMA medium. This process was repeated at least three times (successive passages) until pure cultures were obtained, verified by: (i) uniformity of colony morphology, (ii) Gram staining and microscopic observation (1,000×, oil immersion), and (iii) consistency in generation time.

Pure isolates were systematically cataloged using alphanumeric codes that included the sample type (nodule: N; soil: S; root: R) and isolate number.

##### 2.3.1.7 Preservation and storage in 30% (v/v) glycerol

For long-term preservation, pure isolates were cultured in liquid TY medium (tryptone 5.0 g L^−1^, yeast extract 3.0 g L^−1^, CaCl₂·2H₂O 0.87 g L^−1^, pH 7.0) by inoculation with a loop from fresh colonies (3–5 days of growth). The cultures were incubated at 28 °C with orbital shaking (150–180 rpm) until reaching the late exponential phase (OD₆₀₀ = 0.6–0.8, approximately 24–48 h depending on the isolate).

To prepare glycerol stocks, 700 μL of bacterial culture was aseptically mixed with 300 μL of 100% sterile glycerol (v/v) in 1.5 mL screw-cap cryovials (Sarstedt), resulting in a final glycerol concentration of 30% (v/v). The samples were homogenized by gentle vortexing (10 s) and immediately frozen at −80 °C in an ultra-freezer (Thermo Scientific). This methodology is suitable for both slow- and fast-growing rhizobia, as well as non-rhizobial bacteria, ensuring cell viability for at least 5 years (Somasegaran and Hoben, 1994; Dilworth, 2016).

Strains were recovered for experimental use by thawing the cryovial at room temperature and inoculating an aliquot into liquid TY medium under standard incubation.

### 2.4 Strain selection workflow

From the 36 sampling units, a total of 93 morphologically distinct, presumptively pure bacterial isolates were recovered on YMA medium and assigned codes of the form JSRA_[N/R/S][n], where N = nodule, R = root and S = rhizosphere soil. These 93 isolates were screened for Cd tolerance in TY broth supplemented with 25, 50, 100, 150, and 200 ppm Cd (see section 2.7 for details of the assay). Of these, 53 isolates grew at one or more of the tested Cd concentrations and were considered Cd-tolerant in a broad sense; among them, 33 isolates grew across all five concentrations and were considered consistently tolerant. From this consistently tolerant subset, 23 isolates with the highest visible growth in liquid medium under Cd exposure were prioritized for 16S rRNA Sanger sequencing at Macrogen Inc. (South Korea); the limitation in the number of isolates sent for sequencing was set by the project budget. Of the 23 sequenced isolates, 12 produced near-full-length 16S rRNA sequences of sufficient quality (≥ 50% high-quality reads after assembly and trimming in Sequencher v.5.4.6) for downstream phylogenetic analysis; the remaining 11 isolates were excluded due to poor sequence quality. These 12 strains constitute the dataset analyzed in the present study. The complete strain set, including the 33 consistently Cd-tolerant isolates and the broader 93-isolate collection, is maintained in 30% (v/v) glycerol at −80 °C at the INDES-CES laboratory (Chachapoyas) and is available for follow-up functional evaluation. Taxonomic identification was based on a single near-full-length 16S rRNA marker (universal primers fD1/rD1); we acknowledge that for several genera reliable species-level delimitation would require multilocus sequence analysis (MLSA) or whole-genome sequencing, which lay beyond the scope and budget of the present study (see section 4.8 on limitations).

### 2.5 Bacterial DNA extraction

#### 2.5.1 Rationale and method selection

Molecular taxonomic identification of bacterial isolates was based on sequencing of the 16S rRNA phylogenetic marker gene, widely used for characterization of rhizobia and non-rhizobial bacteria due to its ubiquity in prokaryotes, mosaic structure (conserved and variable regions), and extensive availability of reference sequences in public databases (Yarza et al., 2014; Parte et al., 2020).

For genomic DNA extraction, the thermal lysis method in TE (Tris-EDTA) buffer was used, modified from Moore et al. (2008), selected for its speed, low cost, absence of toxic reagents (phenol-chloroform), and suitability for PCR amplification of 16S rRNA amplicons. Although this method produces DNA of lower purity than commercial column-based kits, it is sufficient for single-locus PCR applications.

#### 2.5.2 Thermal lysis extraction protocol

Genomic DNA was extracted from fresh bacterial cultures (24–48 h, TY medium). A small aliquot of compacted biomass from isolated colonies was transferred to 0.2 mL PCR tubes containing 100 μL of 1 × TE lysis buffer (10 mM Tris–HCl, pH 8.0, 1 mM EDTA).

The suspension was homogenized and placed in a thermocycler under the following thermal-lysis program: lysis at 100 °C for 25 min, snap-cooling at −20 °C for 15 min, and thawing at room temperature for 5–10 min.

The lysed samples were centrifuged at 14,000 × *g* for 10 min at 4 °C in a refrigerated microcentrifuge to pelletize cellular debris and denatured proteins. The supernatant (approximately 90 μL) was carefully pipetted into new, sterile 1.5 mL Eppendorf tubes, taking care to avoid carrying over the pellet. The extracted genomic DNA was stored at −20 °C until use in PCR or at −80 °C for long-term storage (>6 months).

#### 2.5.3 Quality control of the extracted DNA

The integrity of extracted genomic DNA was verified by 0.8% agarose gel electrophoresis in 1 × TAE buffer, with SYBR Safe staining at 1:10,000 dilution.

Electrophoresis was run at 80 V for 30–40 min in 1 × TAE using a standard molecular-weight ladder. High-quality genomic DNA was identified as a diffuse high-molecular-weight band (>10 kb) without smear, and gels were documented with a blue-light transilluminator.

### 2.6 Molecular identification and phylogenetic analysis

Molecular taxonomic identification of bacterial isolates was performed by amplification and sequencing of the 16S rRNA gene, a universal phylogenetic marker widely used due to its ubiquity in prokaryotes and the availability of extensive reference databases (Weisburg et al., 1991; Yarza et al., 2014). The universal primers fD1 (5′-AGAGTTTGATCCTGGCTCAG-3′) and rD1 (5′-AAGGAGGTGATCCAGCC-3′) were used, designed to amplify nearly complete sequences (~1,500 bp) of the 16S rDNA gene in most bacterial genera (Weisburg et al., 1991).

PCR reactions were performed in a final volume of 25 μL using a commercial 2 × PCR master mix (containing Taq DNA polymerase, dNTPs at 400 μM each, MgCl₂ at 3 mM and reaction buffer), with 0.2 μM of each primer and ~5–10 ng of template DNA. The amplification program was: initial denaturation at 94 °C for 3 min; 30 cycles of 94 °C for 50 s, 57 °C for 50 s, 72 °C for 1 min; final extension at 72 °C for 7 min. PCR products were verified on 1.5% agarose gels stained with SYBR Safe, run in 1 × TAE at 75 V for 45 min using a standard molecular-weight ladder. Amplicons of the expected size (~1,500 bp) were column-purified and sent for bidirectional Sanger sequencing at a commercial provider (Macrogen Inc., South Korea).

The raw forward and reverse sequences were processed using Sequencher v.5.4.6 (Gene Codes Corporation, Ann Arbor, MI, United States), a software widely used for the assembly and editing of Sanger sequences due to its efficiency in generating high-quality consensus sequences. The program enabled automatic trimming of low-quality ends, removal of vectors and primer sequences, and assembly of complementary reads into consensus sequences. Taxonomic assignment was performed using BLASTn against the NCBI (GenBank) 16S ribosomal RNA sequences database and LPSN—List of Prokaryotic Names with Standing in Nomenclature, following the identity criteria established by Yarza et al. (2014): ≥99% identity for species level, 95–97% for genus, and 90–95% for family. The sequences of the isolates, together with reference sequences from type strains, were aligned using MAFFT v.7 (Katoh and Standley, 2013) on a HPC (High-Performance Computing) platform, and the phylogenetic trees were inferred using Maximum Likelihood in IQ-TREE 2 (Minh et al., 2020) with automatic selection of the optimal nucleotide substitution model using ModelFinder based on the BIC criterion. Branch support was assessed using ultrafast bootstrap (UFBoot) with 1,000 replicates. Phylogenetic trees were visualized and edited in iTOL v.6 (Interactive Tree of Life) (Letunic and Bork, 2024). The generated sequences were deposited in GenBank under accession numbers PZ012327–PZ012338.

### 2.7 Analysis of genetic diversity using BOX-PCR

The intraspecific genetic diversity of the bacterial isolates was assessed by rep-PCR using the BOX A1R primer (5′-CTACGGCAAGGCGACGCTGACG-3′), a genomic fingerprinting technique (Versalovic et al., 1994) that amplifies intergenic regions flanked by BOX-type repetitive elements, generating strain-specific banding patterns that allow for high genetic discrimination in pathogenic, endophytic, and rhizospheric bacteria (Versalovic et al., 1994; Rademaker and De Bruijn, 1997; Jena et al., 2021; Siddique et al., 2025).

PCR reactions were performed in a final volume of 25 μL containing: GoTaq Green Master Mix 2 × (12.5 μL; Promega), 20 μM BOX A1R primer (1.25 μL, final concentration 1.0 μM), template DNA (~20–50 ng, 2.0 μL), and nuclease-free water (9.25 μL). The amplification program consisted of: initial denaturation at 95 °C for 7 min; 35 cycles of 94 °C for 1 min, 53 °C for 1 min, and 65 °C for 8 min; final extension at 65 °C for 16 min (Rademaker and De Bruijn, 1997).

The amplified products were resolved by electrophoresis in 2.0% (w/v) agarose gel stained with SYBR Safe (Invitrogen) at a 1:10,000 dilution, loading 8 μL of PCR product per lane. GeneRuler 1 kb DNA Ladder (Thermo Scientific) was used as a size reference. Electrophoresis was performed on a runVIEW system (Cleaver Scientific) at 60 V for 4–5 h to maximize band resolution.

The electrophoretic profiles were digitized and analyzed using GelJ v.2.0 software (Heras et al., 2015). The band patterns were converted into a binary presence (1)/absence (0) matrix for each analyzed strain, considering only reproducible and clearly defined bands. Genetic similarity between strains was quantified using the Dice coefficient (Dice, 1945), calculated as:


S_Dice=2a/(2a+b+c).

where a is the number of bands shared between two strains, b is the number of bands present only in the first strain, and c is the number of bands present only in the second strain. Based on the similarity values, the genetic distance matrix was calculated as D = 1 − S_Dice.

Clustering patterns were visualized using a dendrogram constructed by the UPGMA method (Sneath and Sokal, 1973). Given the small number of isolates analyzed (*n* = 12), we did not apply a formal cluster-number optimization procedure. The dendrogram is used here only as a visual aid for grouping isolates by BOX-PCR similarity; the four-cluster structure shown is reported descriptively, without any associated ecological or phylogenetic interpretation.

Genotypic diversity was quantified using the following indices: (i) Shannon index (H′ = −*Σ* p_i × ln p_i), where p_i is the proportion of genotype i in the population (Shannon and Weaver, 1949); (ii) Simpson’s index (D = 1 − Σ p_i^2^), where values close to 1 indicate high diversity (Simpson, 1949); (iii) genotypic richness, defined as the total number of unique genotypes detected; and (iv) percentage of polymorphism, calculated as the proportion of polymorphic bands relative to the total number of bands detected.

Diversity analyses and dendrogram construction were performed in R v.4.5.2 (R Core Team, 2025) using the packages vegan v. 2.7–2 (Oksanen et al., 2025) for calculating the Dice coefficient and diversity indices, and cluster v. 2.1.8.1 (Maechler et al., 2025) for clustering analysis, ape v.5.8 (Paradis and Schliep, 2019) for tree manipulation, ggtree v.3.12.0 (Yu et al., 2017) for dendrogram visualization, dendextend v.1.17.1 (Galili, 2015) for dendrogram editing and ggplot2 v.4.0.0 (Wickham et al., 2025) for graphical visualization.

### 2.8 *In vitro* cadmium tolerance assays

Cadmium tolerance of bacterial isolates was assessed using growth assays in TY liquid medium (tryptone 5.0 g L^−1^, yeast extract 3.0 g L^−1^, CaCl₂·2H₂O 0.87 g L^−1^, pH 7.0) supplemented with increasing concentrations of cadmium. Cadmium chloride monohydrate (CdCl₂·H₂O, analytical grade, HIMEDIA) was used at final concentrations of 0 (control), 25, 50, 100, 150, and 200 mg Cd L^−1^ (ppm). Stock solutions of CdCl₂ (1,000 ppm) were prepared in distilled water, adjusted to pH 7.0 ± 0.2, and sterilized by autoclaving (121 °C, 15 psi, 20 min), following heavy metal tolerance protocols adapted from Gonzalez-Chavez et al. (2002) and methodologies for evaluating cadmium tolerance in rhizobia described by Feria-Cáceres et al. (2022), Matsuda et al. (2002) and Shen et al. (2023).

Isolates stored in 30% (v/v) glycerol at −80 °C were reactivated by plating on YMA medium supplemented with Congo Red (0.0025% w/v) and incubation at 28 °C for 3–5 days. Isolated colonies were inoculated into liquid TY medium and incubated at 28 °C with orbital shaking (150–180 rpm) until the exponential phase was reached (OD₆₀₀ = 0.4–0.6). The cultures were standardized to OD₆₀₀ = 0.1 by dilution with fresh sterile TY medium.

Aliquots of 100 μL of the standardized culture were inoculated into separate test tubes containing 5 mL of TY medium supplemented with different concentrations of Cd, including a cadmium-free control (0 ppm). Three biological replicates were used for each strain-concentration combination (*n* = 3), totaling 216 experimental units (12 strains × 6 concentrations × 3 replicates). The tubes were incubated at 28 °C with orbital shaking at 150 rpm.

Bacterial growth was quantified by measuring the optical density at 600 nm (OD₆₀₀) at 24 and 48 h of incubation, using a UV–Vis spectrophotometer (Eppendorf BioSpectrometer basic). Prior to each reading, the cultures were homogenized by vortexing for 5 s. Uninoculated TY medium was used as a reference blank for each Cd concentration.

The following tolerance parameters were calculated from the OD₆₀₀ data:

Tolerance Index (TI), calculated as the ratio of growth in the presence of Cd to the growth of the metal-free control of the same strain (Zhai et al., 2017):


$$\mathrm{TI}={\mathrm{OD}}_{600}\;\text{treatment}/{\mathrm{OD}}_{600}\;\text{control}\;(\text{same strain}).$$

where OD₆₀₀ control corresponds to the average of measurements of the same strain at 0 ppm Cd. Each strain was evaluated against its own control, given that basal growth rates differ significantly among isolates (range of OD₆₀₀ control: 0.395–0.649). IT values > 1.0 indicate growth stimulation (hormesis) relative to the control (Calabrese and Baldwin, 2003).

Percentage of growth inhibition, calculated as:


Inhibition(%)=[1−TI]×100.

where negative values indicate hormesis.

Median inhibitory concentration (IC₅₀), defined as the concentration of Cd that produces a 50% reduction in OD₆₀₀ relative to the control, estimated for each strain using a four-parameter log-logistic model (LL.4) with the R package drc v.3.0–1 (Ritz et al., 2015):


Y=c+(d−c)/[1+exp(b(log(x)−log(e)))].

where Y is the observed response (OD₆₀₀), x is the cadmium concentration, b is the slope at the inflection point, c is the lower asymptotic limit, d is the upper asymptotic limit, and e is the estimated IC₅₀. When the LL.4 model failed to converge, the LL.3 model (three parameters, with the lower limit set to zero) was used as an alternative. The 95% confidence intervals were calculated using the delta-method.

#### 2.8.1 Statistical analysis

Descriptive statistics (mean, standard deviation, standard error, variance, 95% confidence interval) were calculated by strain and concentration. Assumptions of normality were verified using the Shapiro–Wilk test, and homogeneity of variances using Levene’s test. Since the assumptions of normality were not met in most groups, the nonparametric Kruskal-Wallis test was applied to evaluate the effect of Cd concentration on the OD₆₀₀ of each strain. *Post-hoc* multiple comparisons were performed using the Dunn test with Benjamini-Hochberg correction to control the false discovery rate (*p* < 0.05), implemented using the packages dunn.test v.1.3.6 (Dinno, 2024) and FSA v.0.10.0 (Ogle et al., 2025). The correlation between IC₅₀ and average TI was assessed using Spearman’s correlation coefficient (*ρ*). All analyses were performed in R v.4.5.2 (R Core Team, 2025) using the packages drc v.3.0–1 (Ritz et al., 2015), ggplot2 v.4.0.0 (Wickham et al., 2025), pheatmap v.1.0.13 (Kolde, 2025), and dplyr v.1.1.4 (Wickham et al., 2023).

### 2.9 Functional classification of cadmium tolerance

The functional response of the isolates to cadmium was evaluated using two complementary classification systems based on growth and tolerance indicators obtained *in vitro*, following the functional categorization approach by tolerance levels described by Zhai et al. (2017) for bacteria exposed to cadmium.

The first system was based on the average tolerance index (TI prom), calculated as the mean TI across the five evaluated Cd concentrations (25–200 ppm) and the two measurement times (24 and 48 h). For descriptive purposes only, strains were grouped into four bins defined by the quartiles (Q25, Q50, Q75) of the average TI distribution observed in the set of isolates (range: 0.19–0.64): (i) higher-TI bin (TI ≥ Q75 = 0.55), (ii) intermediate-high TI bin (Q50 ≤ TI < Q75; 0.48–0.55), (iii) intermediate-low TI bin (Q25 ≤ TI < Q50; 0.34–0.48), and (iv) lower-TI bin (TI < Q25 = 0.34). We emphasize that these labels are descriptive statistical bins, not biologically discrete functional categories: strains with closely adjacent TI values (e.g., 0.547 vs. 0.504) may fall into different bins without reflecting a meaningful physiological difference. We therefore use terms such as “higher-TI” or “lower-TI” only as shorthand for position within the empirical TI distribution of this isolate set, and not as intrinsic biological categories. Comparable quartile-based descriptive groupings have been used in previous bacterial Cd-tolerance studies (Matsuda et al., 2002; Feria-Cáceres et al., 2022; Shen et al., 2023).

The second system was based on IC₅₀ values estimated using dose–response models. Strains were classified into five categories: (i) extremely sensitive (IC₅₀ < 10 ppm), (ii) very sensitive (IC₅₀ 10–50 ppm), (iii) sensitive (IC₅₀ 50–100 ppm), (iv) moderately tolerant (IC₅₀ 100–150 ppm), and (v) highly tolerant (IC₅₀ > 150 ppm).

In addition, hormetic responses—defined as TI values > 1.0 (equivalent to TI > 100%)—were identified at one or more Cd concentrations, indicating growth stimulation at sub-toxic doses of the metal. The hormesis phenomenon in microorganisms exposed to heavy metals has been documented as physiological overcompensation mediated by the activation of antioxidant systems and metal homeostasis mechanisms (Calabrese and Baldwin, 2003; Agathokleous et al., 2022).

The possible association between the source of the isolate (*Inga* spp. nodules, *Quararibea* spp. roots, rhizosphere soil) and taxonomic affiliation with the Cd tolerance pattern was explored. The isolates exhibiting the most favorable functional profiles, combining high physiological tolerance (TI ≥ 0.55) and high IC₅₀ values, were identified as priority candidates for further studies aimed at characterizing molecular tolerance mechanisms, evaluating Cd biosorption and bioaccumulation capacities, and developing bioinoculants for contaminated cocoa soils.

The OD₆₀₀ profiles of the set of strains were visualized using a heatmap with hierarchical clustering (Ward. D2 method, Euclidean distance) using the pheatmap v.1.0.13 package (Kolde, 2025). Rows (strains) and columns (concentrations) were clustered independently. The annotation sidebar indicates the source of isolation for each strain.

## 3 Results

### 3.1 Genomic diversity of the isolates (BOX-PCR)

Genomic fingerprinting analysis using BOX-PCR generated polymorphic banding patterns for the 12 bacterial strains evaluated. Amplification with the BOXA1R primer produced a total of 33 bands, 100% of which were polymorphic (Table 2). The number of bands per strain ranged from 4 to 8, with a mean richness of 5.67 ± 1.23 bands (95% CI: 4.97–6.36). The 12 isolates generated unique genotypes (proportion = 100%), confirming that each strain represents a distinct genotype.

**Table 2:** BOX-PCR genotypic diversity indices of bacterial strains isolated from cocoa agroforestry systems in Bagua, Amazonas.

Metric	Value
Number of strains	12
Total bands detected	33
Polymorphic bands (%)	33 (100%)
Mean band richness (± SD)	5.67 ± 1.23
Range of bands/strain	4–8
Unique genotypes (%)	12 (100%)
Shannon index (H′)	2.485
Evenness (J)	1.000
Simpson’s index (D)	0.917
Inverse Simpson (1/D)	12.0
Dice minimum similarity (%)	0.0
Maximum dice similarity (%)	80.0
Maximum similarity pair	N05 – N31
Number of clusters (UPGMA)	4

Genomic profiles generated using BOX-PCR with the BOXA1R primer. Genetic similarity calculated using the Dice coefficient (Dice, 1945). Dendrogram constructed using UPGMA with a cutoff threshold of 98%. Diversity indices calculated in R v.4.5.2 using the vegan package v.2.7–2 (Oksanen et al., 2025). H′ = Shannon index (Shannon and Weaver, 1949); J = Evenness; D = Simpson index (Simpson, 1949). DE, standard deviation.

Diversity indices reflected a population with maximum evenness: Shannon H′ index = 2.485 (Evenness J = 1.000) and Simpson D index = 0.917 (inverse 1/D = 12.0), values equivalent to the maximum theoretical diversity for a population of 12 evenly distributed genotypes.

**Figure 2:**
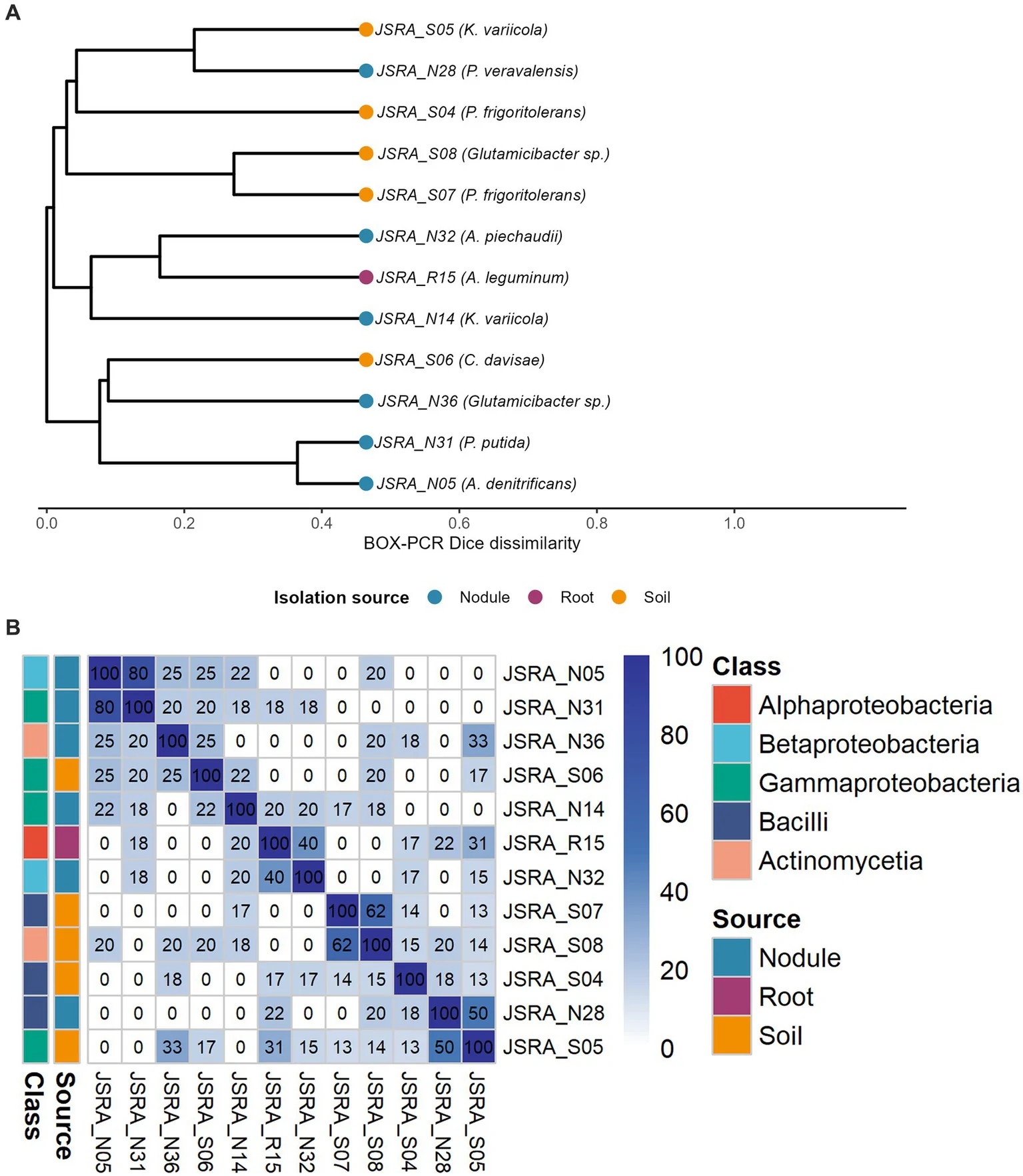
UPGMA dendrogram and Dice similarity matrix based on BOX-PCR genomic fingerprinting of 12 bacterial strains isolated from cacao agroforestry systems in Bagua, Amazonas. **(A)** Dendrogram constructed using the UPGMA method based on the Dice similarity coefficient. Colored circles at tips indicate isolation source: blue (nodule), pink (root), orange (soil). Scale bar represents Dice genetic distance. **(B)** Heatmap of pairwise Dice similarity values (%). Lateral annotation bars indicate isolation source and phylogenetic affiliation. Analysis performed in R v.4.5.2.

The genetic similarity matrix based on the Dice coefficient revealed values ranging from 0 to 80%. The highest similarity was observed between JSRA_N05 and JSRA_N31 (80%), both isolated from *Inga* sp. nodules but belonging to different phyla (Betaproteobacteria and Gammaproteobacteria, respectively). The minimum similarity (0%) was recorded in multiple pairs of strains, indicating the absence of shared bands between genetically distant isolates (Figure 2B).

The UPGMA dendrogram (Figure 2A) allowed the identification of four main clusters. Cluster I grouped the strains JSRA_N05 (Betaproteobacteria), JSRA_N31 (Gammaproteobacteria), JSRA_N36 (Actinobacteria), and JSRA_S06 (Gammaproteobacteria). Cluster II included JSRA_N14 (Gammaproteobacteria), JSRA_R15 (Alphaproteobacteria), and JSRA_N32 (Betaproteobacteria). Cluster III comprised JSRA_N28 (Firmicutes), JSRA_S04 (Firmicutes), and JSRA_S05 (Gammaproteobacteria). Cluster IV comprised JSRA_S07 (Firmicutes) and JSRA_S08 (Actinobacteria). The distribution of strains across clusters did not strictly correspond to isolation source or taxonomic affiliation (Table 3), suggesting that BOX-PCR genomic profiles reflect genomic variability independent of 16S rRNA-based phylogeny.

**Table 3:** BOX-PCR cluster assignment, source of isolation, and taxonomic affiliation of the 12 bacterial strains.

Strain	Cluster	Origin	Host	No. of bands	Phylum/class
JSRA_N05	I	Nodule	*Inga* sp.	5	Betaproteobacteria
JSRA_N31	I	Nodule	*Inga* sp.	6	Gammaproteobacteria
JSRA_N36	I	Nodule	*Inga* sp.	5	Actinobacteria
JSRA_S06	I	Soil	*Inga* sp.	4	Gammaproteobacteria
JSRA_N14	II	Nodule	*Inga* sp.	6	Gammaproteobacteria
JSRA_R15	II	Root	*Quararibea* sp.	5	Alphaproteobacteria
JSRA_N32	II	Nodule	*Inga* sp.	5	Betaproteobacteria
JSRA_N28	III	Nodule	*Inga* sp.	4	Firmicutes
JSRA_S04	III	Soil	*Inga* sp.	7	Firmicutes
JSRA_S05	III	Soil	*Inga* sp.	8	Gammaproteobacteria
JSRA_S07	IV	Soil	*Inga* sp.	7	Firmicutes
JSRA_S08	IV	Soil	*Quararibea* sp.	6	Actinobacteria

Clusters defined using UPGMA with Dice coefficient (98% similarity threshold). No. of bands = number of BOX-PCR bands detected per strain.

### 3.2 Taxonomic identification and phylogenetic diversity

**Figure 3:**
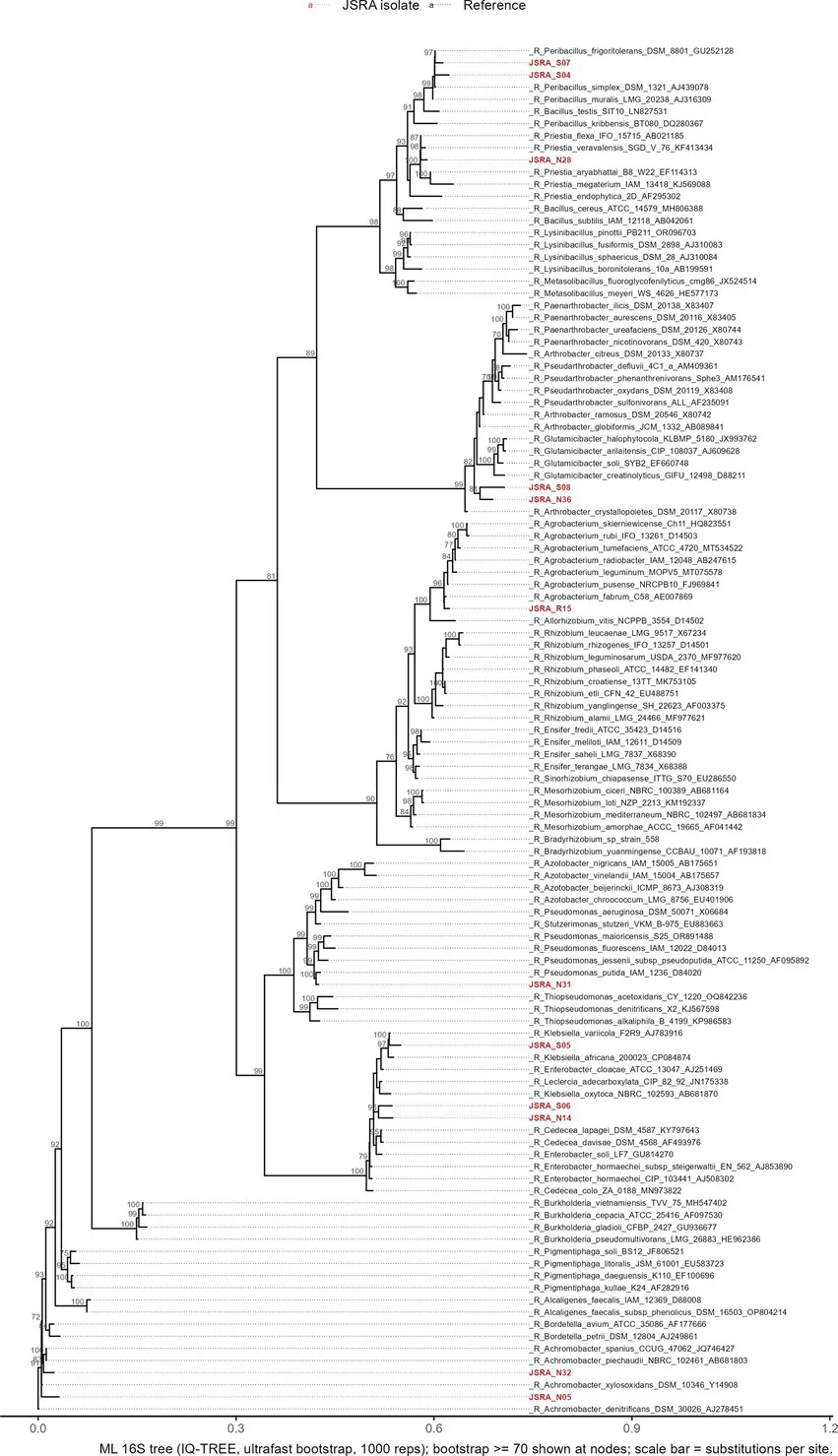
Maximum likelihood phylogenetic tree based on 16S rRNA gene sequences of 12 bacterial strains isolated from cacao agroforestry systems in Bagua, Amazonas. Tree inferred using IQ-TREE 2 with automatic model selection via ModelFinder (BIC criterion). Circle size is proportional to ultrafast bootstrap support (1,000 replicates; scale: 19–100). Study strains are highlighted (JSRA prefix). Note the absence of clustering with classical nitrogen-fixing rhizobia despite six strains being isolated from *Inga* sp. nodules. Tree visualized in iTOL v.6. GenBank accession numbers: PZ012327–PZ012338.

Molecular identification via 16S rRNA gene sequencing and maximum likelihood phylogenetic analysis (IQ-TREE 2, 1,000 ultrafast bootstrap replicates) revealed that the 12 isolates were distributed across five taxonomic groups belonging to three bacterial phyla: Proteobacteria (classes Alpha-, Beta-, and Gammaproteobacteria), Firmicutes, and Actinobacteria (Figure 3 and Table 4). The sequences were deposited in GenBank under accession numbers PZ012327–PZ012338.

**Table 4:** Taxonomic identification by 16S rRNA phylogeny and BOX-PCR cluster assignment.

Strain	Cluster	Phylum/class	Closest species (16S)	Bootstrap	GenBank accession no.
JSRA_N05	I	Betaproteobacteria	*Achromobacter denitrificans*	91ᵃ	PZ012327
JSRA_N14	II	Gammaproteobacteria	*Klebsiella variicola*	96	PZ012328
JSRA_R15	II	Alphaproteobacteria	*Agrobacterium leguminum*	96	PZ012329
JSRA_N28	III	Firmicutes	*Priestia veravalensis*	98	PZ012330
JSRA_N31	I	Gammaproteobacteria	*Pseudomonas putida*	100	PZ012331
JSRA_N32	II	Betaproteobacteria	*Achromobacter piechaudii*	83	PZ012332
JSRA_N36	I	Actinobacteria	*Arthrobacter/Glutamicibacter* sp.	81	PZ012333
JSRA_S04	III	Firmicutes	*Peribacillus frigoritolerans*	99	PZ012334
JSRA_S05	III	Gammaproteobacteria	*Klebsiella variicola*	100	PZ012335
JSRA_S06	I	Gammaproteobacteria	*Cedecea davisae*	79	PZ012336
JSRA_S07	IV	Firmicutes	*Peribacillus frigoritolerans*	97	PZ012337
JSRA_S08	IV	Actinobacteria	*Arthrobacter/Glutamicibacter* sp.	81	PZ012338

Phylogeny inferred using IQ-TREE 2 (maximum likelihood) based on 16S rRNA sequences (~1,500 bp, fD1/rD1 primers). Bootstrap = ultrafast bootstrap support (1,000 replicates). ᵃ Bootstrap corresponding to the Achromobacter clade to which the strain belongs; the immediate node has a support of 72.

A note on taxonomic resolution. Taxonomic assignments are based on a single near-full-length 16S rRNA marker (universal primers fD1/rD1). The 16S rRNA gene is reliable for assignment at genus level and above, but for several of the genera recovered here (in particular *Klebsiella, Pseudomonas, Achromobacter, Cedecea and Glutamicibacter*) reliable species-level delimitation requires multilocus sequence analysis (MLSA) or whole-genome sequencing, which were beyond the scope and budget of the present study. Throughout this paper, species-level names (e.g., *Klebsiella variicola, Pseudomonas putida, Cedecea davisae*, *Achromobacter denitrificans*, *Achromobacter piechaudii*, *Agrobacterium leguminum, Priestia veravalensis, Peribacillus frigoritolerans*) should be read as denoting the closest type-strain match in the 16S rRNA database (BLASTn against NCBI 16S ribosomal RNA sequences and LPSN), and not as a definitive species-level identification. Bootstrap support for the corresponding nodes is reported in Table 4 and Figure 3; nodes with support below 90% [e.g., JSRA_S06 (79%), JSRA_N36 and JSRA_S08 (81%), JSRA_N32 (83%)] should be interpreted especially cautiously and are best regarded as genus-level assignments.

Within Firmicutes (Bacillaceae), three strains were identified. JSRA_N28, isolated from an *Inga* sp. nodule, showed affinity with *Priestia veravalensis* (bootstrap 98%). JSRA_S04 and JSRA_S07, both from the rhizosphere soil of *Inga* sp., were grouped with *Peribacillus frigoritolerans* (bootstrap 99 and 97%, respectively). These endospore-forming genera are known for their metabolic versatility and ability to colonize environments subjected to abiotic stress.

The phylum Actinobacteria (family Micrococcaceae) was represented by two strains: JSRA_N36 (nodule of *Inga* sp.) and JSRA_S08 (soil of *Quararibea* sp.), both identified as *Arthrobacter/Glutamicibacter* sp. (bootstrap 81%). Their presence in both nodules and soil suggests the ability to colonize multiple ecological niches.

Within Gammaproteobacteria, four strains were distributed across two families. In Pseudomonadaceae, JSRA_N31 (*Inga* sp. nodule) was grouped with *Pseudomonas putida* (bootstrap 100%). In Enterobacteriaceae, JSRA_N14 (nodule) and JSRA_S05 (soil) showed affinity with *Klebsiella variicola* (bootstrap 96 and 100%, respectively), while JSRA_S06 (soil) was identified as *Cedecea davisae* (bootstrap 79%).

The class Betaproteobacteria (Alcaligenaceae) was represented by two nodule strains: JSRA_N05, identified as *Achromobacter denitrificans* (*Achromobacter* clade, bootstrap 91%), and JSRA_N32, grouped with *Achromobacter piechaudii* (bootstrap 83%).

Finally, the class Alphaproteobacteria (Rhizobiaceae) was represented by JSRA_R15, isolated from roots of *Quararibea* sp., which was grouped with *Agrobacterium leguminum* (bootstrap 96%). This finding is noteworthy given that *Agrobacterium*, although belonging to Rhizobiaceae, is not a nitrogen-fixing rhizobium sensu stricto.

The most significant finding from an ecological perspective was the absence of rhizobia sensu stricto among the cultivable isolates recovered under the conditions employed (genera *Rhizobium*, *Bradyrhizobium*, *Mesorhizobium*, *Ensifer*) among the 12 isolates, including the six strains obtained directly from root nodules of *Inga* sp. All nodule-derived cultivable isolates corresponded to non-rhizobial endophytic bacteria (NREs) belonging to Firmicutes (*Priestia*), Actinobacteria (*Glutamicibacter*), Betaproteobacteria (*Achromobacter*), and Gammaproteobacteria (*Pseudomonas*, *Klebsiella*). Within the limits of this culture-dependent dataset, the cultivable nodule fraction recovered from *Inga* sp. nodules in Cd-impacted Amazonian cocoa agroforestry systems was composed exclusively of NRE lineages, several of which carry traits previously associated with adaptation to metal stress. Whether classical rhizobia are truly absent or simply not retrieved under these conditions cannot be resolved with the present approach and would require culture-independent confirmation (e.g., amplicon sequencing, metagenomics, or qPCR of symbiotic markers such as *nodA* or *nifH*).

### 3.3 Response of isolates to cadmium

#### 3.3.1 Tolerance index and functional classification

Cd tolerance assays revealed marked functional heterogeneity among the 12 isolates. The average tolerance index (TI prom), calculated using the individual control for each strain, ranged from 0.190 (JSRA_S04) to 0.643 (JSRA_S05), with an average growth inhibition between 35.7 and 81.0% (Table 5).

**Table 5:** Tolerance index (TI) by strain and cadmium concentration, functional classification by quartiles, and IC_50_.

Strain	Origin	Host	TI 25	TI 50	TI 100	TI 150	TI 200	TI prom	TI category	IC₅₀ (ppm)	IC₅₀ category
JSRA_S05	Soil	*Inga* sp.	1.235	1.178	0.390	0.185	0.227	0.643	Tolerant height	93.2	Sensitive
JSRA_S08	Soil	*Quararibea* sp.	0.952	**1.012**	0.457	0.605	0.175	0.640	Tolerant height	124.9	Mod. tolerance
JSRA_R15	Root	*Quararibea* sp.	0.683	0.655	0.427	0.235	0.852	0.570	Tolerant	301.7	Highly tolerant
JSRA_N32	Nodule	*Inga* sp.	0.562	0.658	0.817	0.425	0.275	0.547	Tolerant	131.4	Tolerant mod.
JSRA_N05	Nodule	*Inga* sp.	0.860	0.672	0.379	0.487	0.336	0.547	Tolerant	46.0	Very sensitive
JSRA_N36	Nodule	*Inga* sp.	0.593	0.520	0.610	0.435	0.363	0.504	Tolerant	87.4	Sensitive
JSRA_N31	Nodule	*Inga* sp.	0.468	0.534	0.510	0.433	0.368	0.463	Sensitive	37.1	Very sensitive
JSRA_N28	Nodule	*Inga* sp.	0.429	0.418	0.352	0.302	0.302	0.361	Sensitive	11.8	Ext. sensitive
JSRA_S06	Soil	*Inga* sp.	0.181	0.279	0.288	0.493	0.517	0.352	Sensitive	143.8	Tolerant model
JSRA_S07	Soil	*Inga* sp.	0.194	0.254	0.240	0.221	0.565	0.295	Sensitive Alt.	225.6	Tolerant alt.
JSRA_N14	Nodule	*Inga* sp.	0.099	0.070	0.380	0.177	0.229	0.191	Sensitive Alt.	719.7	Tolerant alt.
JSRA_S04	Soil	*Inga* sp.	0.246	0.199	0.132	0.236	0.135	0.190	Sensitive Alt.	0.18	Ext. sensitive

TI = OD₆₀₀ treatment/OD₆₀₀ control (same strain). Values in bold indicate average TI > 1.0 (interpreted as clear hormesis for JSRA_S05 and as a possible hormetic tendency for JSRA_S08, see Discussion 4.4). Avg. TI = average TI across 5 concentrations and 2 measurement times (*n* = 30 per strain). TI categories by quartiles: highly tolerant (TI ≥ 0.55), tolerant (0.48–0.55), sensitive (0.34–0.48), highly sensitive (< 0.34). IC₅₀ categories: extremely sensitive (< 10 ppm), very sensitive (10–50), sensitive (50–100), moderately tolerant (100–150), highly tolerant (> 150). IC₅₀ estimated using LL.4/LL.3 models (drc package, R).

Quartile classification divided the isolates into four balanced categories (*n* = 3 per category):

The highly tolerant isolates (TI ≥ 0.55; *n* = 3) included JSRA_S05 (TI = 0.643), JSRA_S08 (TI = 0.640), and JSRA_R15 (TI = 0.570). These strains maintained more than 55% of their relative growth across the Cd gradient. Notably, the two strains associated with *Quararibea* sp. (JSRA_S08 and JSRA_R15) fell into this category, along with JSRA_S05 from *Inga* sp. soil.

The tolerant isolates (TI 0.48–0.55; *n* = 3) were JSRA_N32 (TI = 0.547), JSRA_N05 (TI = 0.547), and JSRA_N36 (TI = 0.504), all isolated exclusively from *Inga* sp. nodules and belonging to different phyla (Betaproteobacteria, Betaproteobacteria, and Actinobacteria), suggesting convergent tolerance among phylogenetically distant lineages that coexist in the nodular niche.

The sensitive isolates (TI 0.34–0.48; *n* = 3) included JSRA_N31 (TI = 0.463), JSRA_N28 (TI = 0.361), and JSRA_S06 (TI = 0.352), with representatives of Gammaproteobacteria and Firmicutes from nodules and soil.

**Figure 4:**
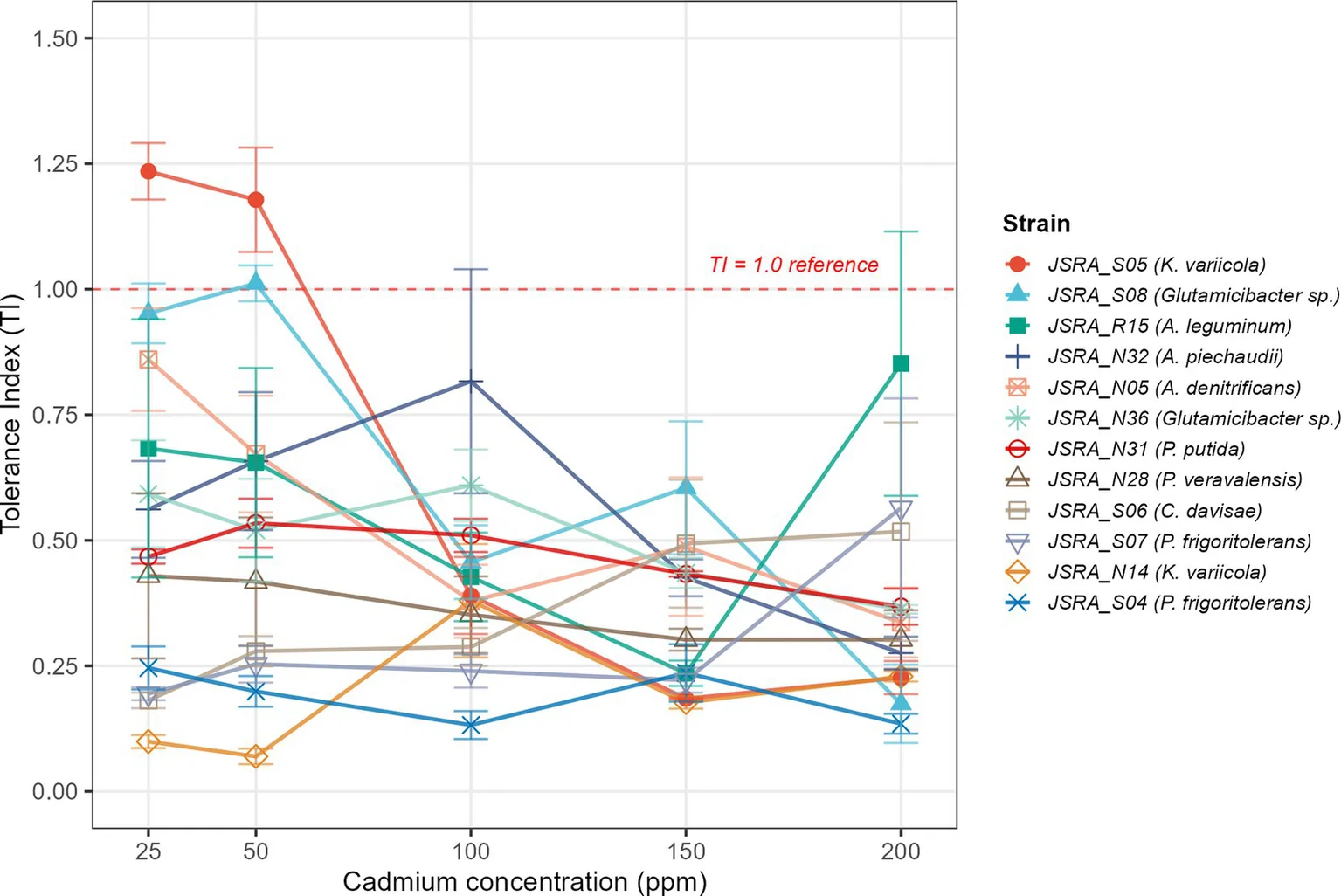
Dose–response curves of tolerance index (TI) across cadmium concentrations for 12 bacterial strains. Each point represents the mean TI of six measurements (3 replicates × 2 time points); error bars indicate standard error. The red dashed line at TI = 1.0 represents the hormesis threshold. Confirmed hormesis was observed for JSRA_S05 at 25 ppm (TI = 1.235) and 50 ppm (TI = 1.178), and for JSRA_S08 at 50 ppm (TI = 1.012). Strains ordered by decreasing mean TI.

The highly sensitive isolates (TI < 0.34; *n* = 3) were JSRA_S07 (TI = 0.295), JSRA_N14 (TI = 0.191), and JSRA_S04 (TI = 0.190), with growth inhibition exceeding 70%, indicating limited physiological adaptability to metal stress (Figure 4).

#### 3.3.2 Hormetic responses

A clear hormetic response (average TI > 1.0 at two consecutive concentrations) was observed in JSRA_S05, with TI = 1.235 at 25 ppm and TI = 1.178 at 50 ppm, indicating growth stimulation of 23.5 and 17.8% relative to the control, respectively. JSRA_S08 showed only a borderline value at 50 ppm (TI = 1.012, i.e., 1.2% above control), which falls within the typical experimental variability of OD₆₀₀-based growth assays and is therefore interpreted here as a possible hormetic tendency rather than as a confirmed hormetic response; formal statistical testing of individual TI values against the corresponding control was not performed, and the value of TI = 1.012 should be interpreted with this caveat. For JSRA_S05, stimulation at low-to-medium concentrations was followed by progressive inhibition at higher concentrations, forming a biphasic dose–response profile consistent with the classic hormetic model (Calabrese and Baldwin, 2003; Agathokleous et al., 2022). Both isolates showing TI > 1.0 at any concentration were recovered from rhizosphere soil, which could reflect greater historical exposure to bioavailable Cd in the soil solution (Supplementary Figure S1).

At the level of individual measurements, TI values > 1.0 were detected in additional strains: JSRA_N05 at 25 ppm (3/6 measurements), JSRA_N32 at 100 ppm (2/6), JSRA_R15 at 25, 50, and 200 ppm (3/6 at each concentration), JSRA_S06 at 200 ppm (1/6), and JSRA_S07 at 200 ppm (2/6). These values were not confirmed as hormesis at the level of concentration averages, reflecting high variability among replicates and measurement times.

#### 3.3.3 Half-maximal inhibitory concentration (IC₅₀)

**Figure 5:**
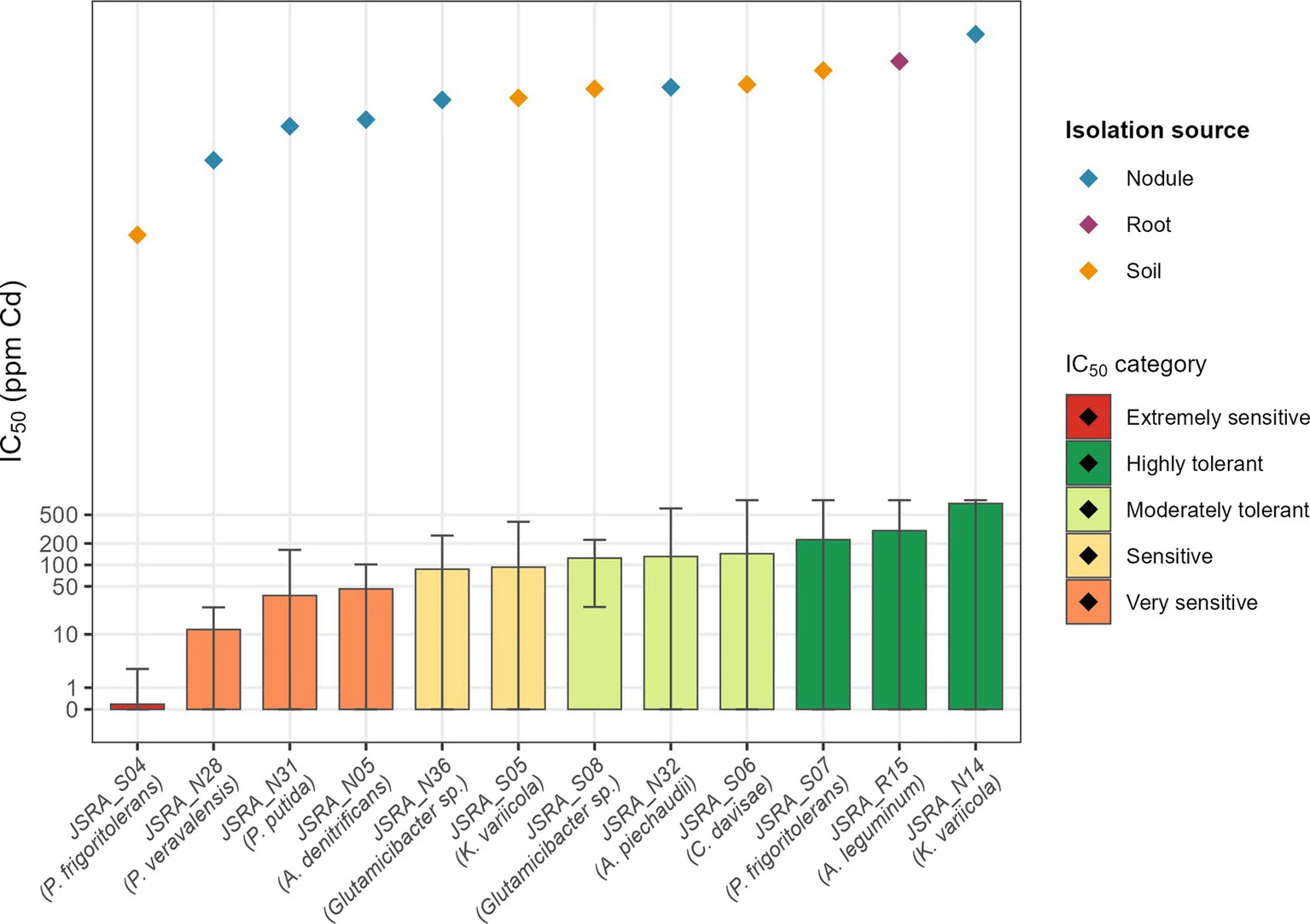
Comparison of half-maximal inhibitory concentration (IC_50_) values among 12 bacterial strains. IC_50_ estimated by log-logistic dose–response models (LL.4 or LL.3). Y-axis on log_10_(1 + *x*) scale. Bar colors indicate IC_50_ category: Red (extremely sensitive, <10 ppm), orange (very sensitive, 10–50), yellow (sensitive, 50–100), light green (moderately tolerant, 100–150), dark green (highly tolerant, >150 ppm). Diamond symbols indicate isolation source. Error bars represent 95% confidence intervals.

The IC₅₀ values estimated using log-logistic models (Table 5; Figure 5) spanned more than three orders of magnitude: from 0.18 ppm (JSRA_S04, model LL.3) to 719.7 ppm (JSRA_N14, model LL.4). Classification by IC₅₀ grouped the isolates into: extremely sensitive (*n* = 1: JSRA_S04), very sensitive (*n* = 3: JSRA_N28, JSRA_N31, JSRA_N05), sensitive (*n* = 2: JSRA_N36, JSRA_S05), moderately tolerant (*n* = 3: JSRA_S08, JSRA_N32, JSRA_S06), and highly tolerant (*n* = 3: JSRA_S07, JSRA_R15, JSRA_N14).

Several models exhibited wide confidence intervals (JSRA_R15: 95% CI = −4756.5 to 5359.8; JSRA_N14: 95% CI not estimable), reflecting the presence of non-monotonic patterns that complicate the fitting of classical log-logistic models. These IC₅₀ values should be interpreted as reference estimates.

#### 3.3.4 Discrepancy between TI and IC₅₀ classifications

A notable discrepancy was observed between classifications based on average TI and IC₅₀ (Table 5). The Spearman correlation between the two parameters was low and not significant (*ρ* < 0.1, *p* > 0.05), indicating that TI and IC₅₀ capture complementary dimensions of cadmium tolerance. The most illustrative cases were: JSRA_N14 (highly sensitive by TI = 0.191, but highly tolerant by IC₅₀ = 719.7 ppm), JSRA_S07 (highly sensitive by TI = 0.295, but highly tolerant based on IC₅₀ = 225.6 ppm), and JSRA_S05 (highly tolerant based on TI = 0.643, but sensitive based on IC₅₀ = 93.2 ppm).

This discrepancy is explained by the fact that the average TI reflects the general ability to maintain growth across the entire Cd gradient, whereas the IC₅₀ estimates the inflection point of the dose–response curve, which may be influenced by non-monotonic patterns. In the case of JSRA_N14, its low average TI reflects severe inhibition at most concentrations, but its high IC₅₀ results from a growth peak at 100 ppm (TI = 0.380) that shifts the fitted curve. These results underscore the importance of using complementary classification systems for a comprehensive assessment of bacterial tolerance to Cd.

#### 3.3.5 Inferential statistical analysis

**Figure 6:**
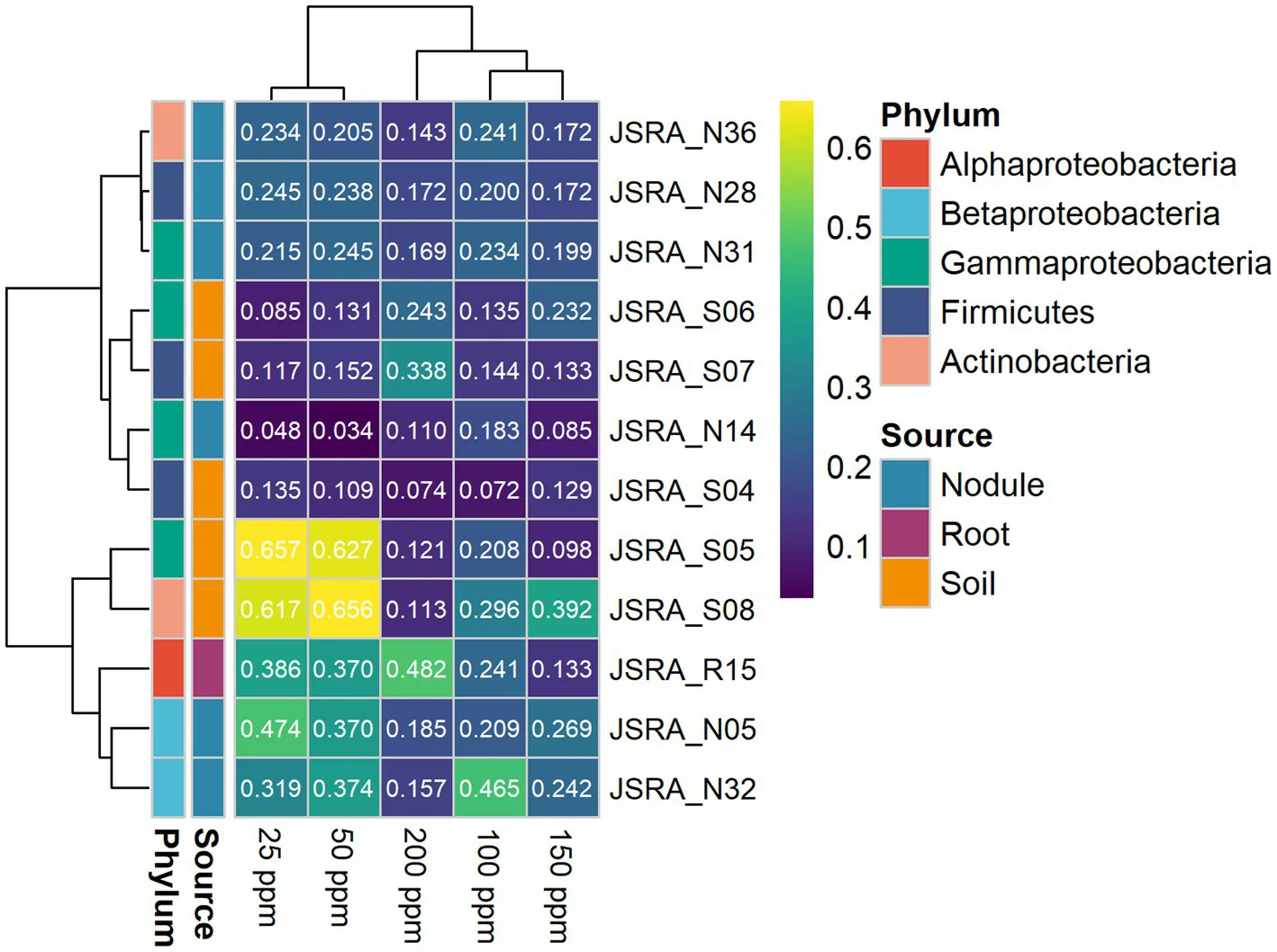
Heatmap of mean OD_600_ values of 12 bacterial strains exposed to increasing cadmium concentrations (25–200 ppm). Color scale: Viridis palette (yellow = high OD_600_, purple = low OD_600_). Hierarchical clustering (Ward. D2, Euclidean distance) applied independently to rows and columns. Lateral annotation bars indicate isolation source and phylogenetic affiliation.

The Kruskal-Wallis test confirmed a significant effect of Cd concentration on OD₆₀₀ in most isolates. Dunn’s *post-hoc* comparisons with Benjamini-Hochberg correction (Figure 6) identified significant differences (*p* < 0.05) primarily between the control (0 ppm) and concentrations ≥ 100 ppm in the highly tolerant strains, and between the control and concentrations ≥ 25 ppm in the highly sensitive strains, confirming the differential sensitivity of the isolates to the Cd gradient.

### 3.4 Taxonomy–function integration

**Figure 7:**
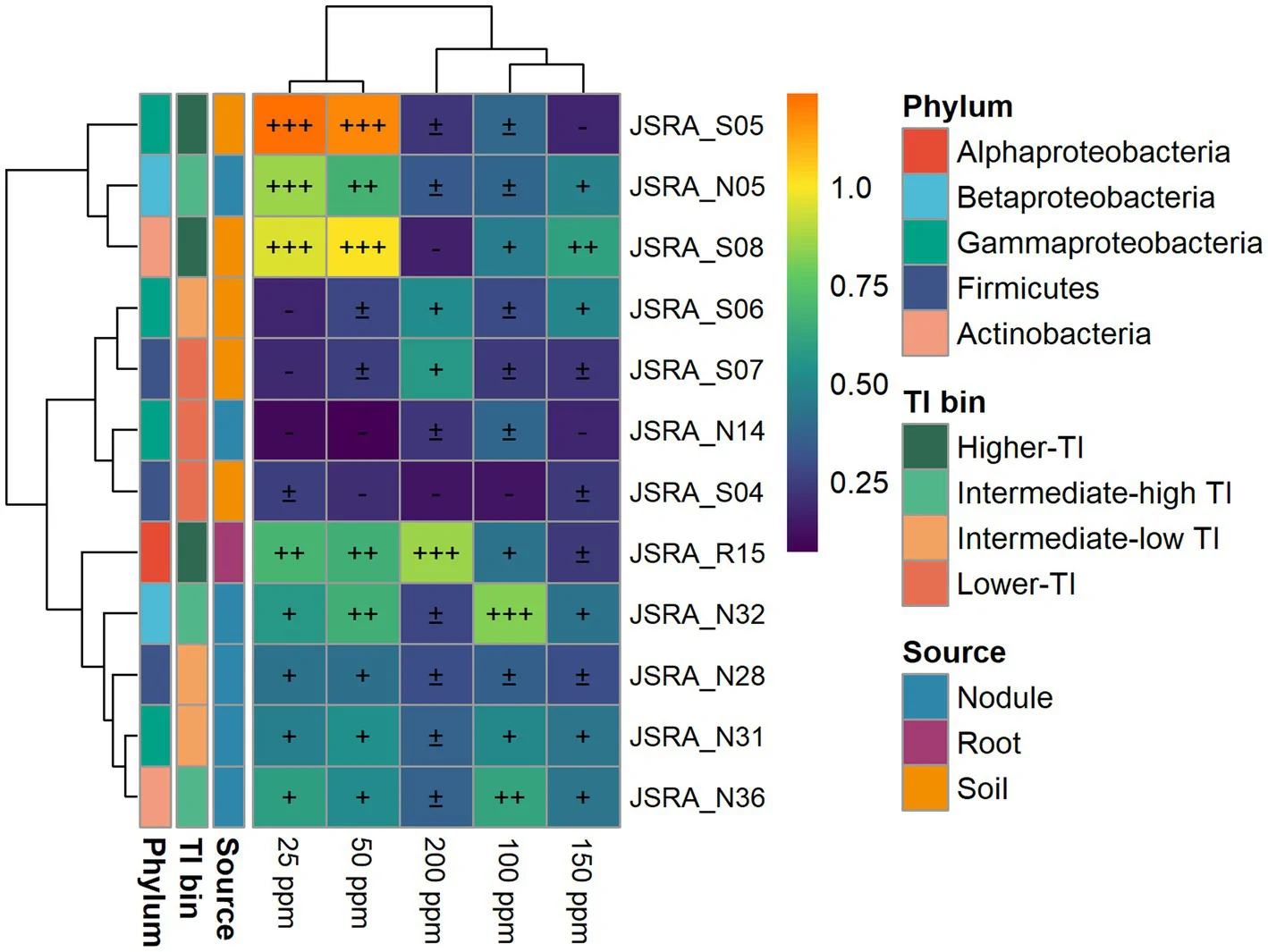
Heatmap of Tolerance Index (*TI*) with tolerance symbols for 12 bacterial strains across cadmium concentrations (25–200 ppm). Symbols: +++ (*TI* ≥ 0.80), ++ (0.60–0.79), + (0.40–0.59), ± (0.20–0.39), − (<0.20). Lateral annotation bars indicate isolation source, *TI* category (quartile classification), and phylogenetic affiliation. Color scale: Purple (low *TI*) to yellow-orange (high *TI*/hormesis).

The integration of phylogenetic identity, isolation origin, and Cd tolerance phenotype revealed ecologically relevant patterns (Figure 7; Table 6).

**Table 6:** Integrated summary of taxonomy, origin, and cadmium tolerance of the 12 bacterial isolates.

Phylum/class	Strain	Closest species	Origin	Host	TI prom	TI category	IC₅₀	Hormesis
Gammaproteobacteria	JSRA_S05	*K. variicola*	Soil	*Inga*	0.643	Alt. tolerant	93.2	Yes
Actinobacteria	JSRA_S08	*Glutamicibacter* sp.	Soil	*Quararibea*	0.640	Alt. tolerant	124.9	Tendency
Alphaproteobacteria	JSRA_R15	*A. leguminum*	Root	*Quararibea*	0.570	Tolerant	301.7	No
Betaproteobacteria	JSRA_N32	*A. piechaudii*	Nodule	*Inga*	0.547	Tolerant	131.4	No
Betaproteobacteria	JSRA_N05	*A. denitrificans*	Nodule	*Inga*	0.547	Tolerant	46.0	No
Actinobacteria	JSRA_N36	*Glutamicibacter* sp.	Nodule	*Inga*	0.504	Tolerant	87.4	No
Gammaproteobacteria	JSRA_N31	*P. putida*	Nodule	*Inga*	0.463	Sensitive	37.1	No
Firmicutes	JSRA_N28	*P. veravalensis*	Nodule	*Inga*	0.361	Sensitive	11.8	No
Gammaproteobacteria	JSRA_S06	*C. davisae*	Soil	*Inga*	0.352	Sensitive	143.8	No
Firmicutes	JSRA_S07	*P. frigoritolerans*	Soil	*Inga*	0.295	Sensitive alt.	225.6	No
Gammaproteobacteria	JSRA_N14	*K. variicola*	Nodule	*Inga*	0.191	Sensitive height	719.7	No
Firmicutes	JSRA_S04	*P. frigoritolerans*	Soil	*Inga*	0.190	Sensitive alt.	0.18	No

Strains sorted by descending average TI. Hormesis = average TI > 1.0 at least one Cd concentration.

Regarding the source of isolation, the two strains associated with *Quararibea* sp. (JSRA_S08 and JSRA_R15) were classified as highly tolerant and tolerant, respectively, and JSRA_R15 exhibited the second-highest IC₅₀ in the study (301.7 ppm). This result suggests that *Quararibea* sp., a species known for its ability to accumulate heavy metals, harbors microorganisms with higher levels of Cd tolerance. Among the *Inga* sp. nodule strains, the greatest functional heterogeneity was observed, with representatives in all four TI categories, ranging from highly tolerant (JSRA_N32, JSRA_N05) to highly sensitive (JSRA_N14).

Regarding taxonomic affiliation, Cd tolerance did not show a defined phylogenetic pattern. Within Firmicutes, the three strains (*Priestia veravalensis* and two *Peribacillus frigoritolerans*) were sensitive or highly sensitive. Within Betaproteobacteria, both *Achromobacter* strains were tolerant. The Gammaproteobacteria strains showed the greatest diversity of responses, ranging from highly tolerant (*K. variicola* JSRA_S05, with hormesis) to highly sensitive (*K. variicola* JSRA_N14), including two strains of the same species with diametrically opposed responses. This intraspecific heterogeneity suggests that Cd tolerance is a strain-specific trait determined by accessory genomic factors rather than by taxonomic identity at the genus or species level.

Candidate strains for future functional evaluation, selected on the basis of high *in vitro* physiological tolerance (TI ≥ 0.55) and complementary functional profiles, include: JSRA_S05 (*Klebsiella variicola*, *Inga* sp. soil, confirmed hormesis), JSRA_S08 (*Glutamicibacter* sp., *Quararibea* sp. soil, possible hormetic tendency), and JSRA_R15 (*Agrobacterium leguminum*, *Quararibea* sp. root, IC₅₀ = 301.7 ppm). These three strains span three different phyla and two compartments (soil and root), features that may be relevant for subsequent functional screening (e.g., biosorption, bioaccumulation, plant inoculation). *In vitro* Cd tolerance demonstrates the capacity to grow in the presence of the metal, but does not by itself establish bioremediation potential; further work is required to assess metal sequestration, immobilization, transformation, effects on plant uptake and field efficacy before any bioinoculant application can be proposed.

## 4 Discussion

### 4.1 Non rhizobial endophytes as the cultivable nodular fraction recovered from *Inga* sp. under cadmium stress

The most notable observation of this study was that no rhizobia sensu stricto (*Rhizobium, Bradyrhizobium, Mesorhizobium, Ensifer*) were recovered among the six cultivable isolates obtained from *Inga* sp. root nodules under the conditions employed, despite the use of YMA medium supplemented with Congo Red, a formulation designed for the presumptive selection of rhizobia (Somasegaran and Hoben, 1994). Instead, all nodule-derived cultivable isolates corresponded to non-rhizobial endophytic bacteria (NREs) belonging to three phyla and four classes: Betaproteobacteria (*Achromobacter*), Gammaproteobacteria (*Pseudomonas, Klebsiella*), Firmicutes (*Priestia*), and Actinobacteria (*Glutamicibacter*). Given the well-known limitations of culture-based recovery for slow-growing rhizobia, particularly bradyrhizobia that typically dominate tropical leguminous trees, this finding is best interpreted as the dominance of NREs within the cultivable fraction, rather than as evidence of true community-level absence of rhizobia (see also section 4.8 on limitations).

This result contrasts with that reported by Eaton et al. (2024), who, using culture-independent high-throughput sequencing, identified *Bradyrhizobium diazoefficiens* as the dominant taxon (63–86% of the sequences) in *Inga* punctata nodules in a Costa Rican cloud forest, accompanied by 26 genera of non-rhizobial bacteria. Two non-mutually exclusive factors may explain this divergence. First, the methodological framework differs substantially: Eaton et al. employed amplicon sequencing, which captures both fast- and slow-growing taxa, whereas the present study is based on culture-dependent recovery on YMA, conditions known to disfavor slow-growing bradyrhizobia. Second, the edaphic context differs: while the Costa Rican soils showed no documented heavy metal contamination, the plots in Bagua recorded Cd concentrations between 1.02 and 3.54 mg kg^−1^, exceeding Peruvian environmental regulations (MINAM, 2017). Whether Cd stress exerts selective pressure on nodular communities, favoring lineages with adaptive strategies against metal stress over classical rhizobia, remains a plausible hypothesis consistent with that proposed by Fagorzi et al. (2018), but cannot be tested with the present culture-dependent dataset and a single sampling region.

Our findings are consistent with the growing evidence that legume nodules harbor complex bacterial communities that transcend the classical rhizobial paradigm. Hassen et al. (2025) reported that between 87 and 99% of the bacterial population in nodules consists of NREs, while Hnini and Aurag (2024) documented the prevalence and diversity of these endophytes in a recent systematic review. Jesus et al. (2023) proposed the concept of nodules as a “guesthouse” for a diverse microbiome in *Acacia longifolia*, suggesting that nodular colonization by NREs is a more widespread phenomenon than previously recognized. Flores-Duarte et al. (2022) demonstrated that *Pseudomonas* sp. isolated from legume nodules can promote plant growth in degraded soils, showing that NREs can assume functional roles complementary to those of rhizobia. These findings, together with the results of the present study, reinforce the need to reconsider the model of rhizobial exclusivity in legume nodules, particularly in agroecosystems subjected to heavy metal stress.

Additionally, it is important to consider that the culture approach used (selective media, aerobic conditions) may have favored the isolation of fast-growing NREs over slow-growing rhizobia, particularly bradyrizobia, which predominate in tropical tree legumes (Etesami and Santoyo, 2025). Complementation with independent cultivation techniques, such as metagenomic sequencing of the 16S rRNA gene directly from nodules, would allow for the assessment of the actual proportion of rhizobia versus NREs in this system.

### 4.2 Multiphyletic taxonomic diversity in the agroforestry system

The distribution of the 12 isolates across three phyla and five bacterial classes reflects remarkable taxonomic diversity for a relatively limited sampling system. The identified phyla—Proteobacteria, Firmicutes, and Actinobacteria—coincide with the dominant bacterial groups reported in Cd-contaminated agricultural soils (Yu et al., 2021) identified Proteobacteria as the dominant phylum in Cd-contaminated arable soils, followed by Firmicutes and Actinobacteria, a pattern consistent with our findings. Feria-Cáceres et al. (2022) isolated genera such as *Klebsiella*, *Enterobacter*, *Bacillus*, and *Cupriavidus* from Cd-contaminated Colombian cocoa soils, several of which coincide with the genera identified in the present study.

Genomic diversity assessed using BOX-PCR (H′ = 2.485, 100% unique genotypes, 100% polymorphism) confirms that each isolate represents a distinct genotype, even though some share taxonomic identification at the species level. The discrepancy between taxonomic identity (16S rRNA) and genomic profile (BOX-PCR) indicates intraspecific genomic variability, a phenomenon widely documented in soil bacteria subjected to environmental stress (Rademaker and De Bruijn, 1997; Siddique et al., 2025). The lack of correspondence between BOX-PCR clusters and phylogenetic affiliation suggests that the repetitive elements amplified by BOX-PCR evolve independently of ribosomal genes, reflecting the differential acquisition of mobile genomic elements associated with adaptation to the local environment.

### 4.3 Functional heterogeneity in the response to cadmium

Tolerance assays revealed a broad spectrum of physiological responses, with average IT ranging from 0.190 to 0.643 and IC₅₀ ranging from 0.18 to 719.7 ppm. This heterogeneity is consistent with what has been reported for bacterial communities in Cd-contaminated soils. Ma et al. (2016b) evaluated Cd-tolerant bacteria isolated from contaminated agricultural soils and reported significant variability in their tolerance capacities and bioremediation potential. Zhai et al. (2017) classified 20 strains of *Lactobacillus plantarum* into four Cd tolerance groups based on MIC (10–50 mg/L ≈ ppm), demonstrating that variability in heavy metal tolerance is a widespread phenomenon in bacterial populations.

The dual classification used in this study (TI by quartiles + IC₅₀) revealed that both parameters capture complementary dimensions of tolerance. The low correlation between TI and IC₅₀ (Spearman *ρ* < 0.1) suggests the presence of nonlinear responses, including hormesis, delayed recovery, and biphasic patterns. The cases of JSRA_N14 (highly sensitive by TI = 0.191, but highly tolerant by IC₅₀ = 719.7 ppm) and JSRA_S05 (highly tolerant by TI = 0.643, but sensitive based on IC₅₀ = 93.2 ppm) illustrate how the average TI reflects the general ability to maintain growth across the Cd gradient, while the IC₅₀ estimates the inflection point of the curve, which may be influenced by non-monotonic patterns. The wide confidence intervals observed in several IC₅₀ values reflect the limitation of the log-logistic model in describing complex dose–response curves (Ritz et al., 2015). This observation underscores the importance of using complementary classification systems for a comprehensive assessment of bacterial tolerance to Cd.

### 4.4 Bacterial cadmium hormesis: ecological significance

The clear hormetic response observed in JSRA_S05 (*K. variicola*, TI = 1.235 at 25 ppm), together with the borderline TI value observed in JSRA_S08 (*Glutamicibacter* sp., TI = 1.012 at 50 ppm, interpreted as a possible hormetic tendency within experimental variability), represent, to the best of our knowledge, the first description of biphasic Cd dose–response in cultivable bacteria from Amazonian cocoa agroforestry systems. Hormesis, defined as the stimulation of growth at sub-toxic doses of a stressor, has been documented in various microorganisms exposed to heavy metals (Calabrese and Baldwin, 2003) and recently contextualized at the level of microbial populations by Agathokleous et al. (2022), who proposed that hormesis in microorganisms reflects physiological overcompensation mechanisms, including the activation of antioxidant systems and metal homeostasis. We acknowledge that for JSRA_S08 the 1.2% increase relative to control falls within typical OD₆₀₀ assay variability and was not subjected to formal statistical testing against the control; this isolate is therefore better described as showing a tendency consistent with hormesis rather than confirmed hormesis.

For JSRA_S05 the observed biphasic profile—stimulation at 25–50 ppm followed by progressive inhibition at ≥ 100 ppm—is consistent with the classic hormetic model of inverted U-shaped dose–response (Calabrese and Baldwin, 2003). Both isolates with TI > 1.0 at any concentration were recovered from rhizosphere soil; this could be tentatively interpreted as a sign that chronic exposure to bioavailable Cd in the soil solution may select for more efficient detoxification mechanisms than those present in protected endophytic compartments, although the limited number of isolates with this pattern (*n* = 2) does not allow firm conclusions.

From an applied perspective, isolates showing growth that is at least maintained (and possibly stimulated) at environmentally relevant concentrations of Cd are interesting candidates for future functional evaluation, considering that soils in the Bagua agroforestry area have been characterized with 1.02–3.54 mg Cd kg^−1^ (Table 1).

### 4.5 Strain-specific tolerance: the case of *Klebsiella variicola*

One of the most relevant findings was the diametrically opposed response to Cd of two strains identified as *K. variicola*: JSRA_S05 (highly tolerant, TI = 0.643, confirmed hormesis) and JSRA_N14 (highly sensitive, TI = 0.191). Both share taxonomic identity based on 16S rRNA but exhibit completely different BOX-PCR profiles (Dice similarity = 0%) and were isolated from different compartments (soil vs. nodule).

This intraspecific heterogeneity is consistent with reports by Pagnucco et al. (2023), who documented significant variability in tolerance and biosorption of heavy metals among bacterial strains isolated from an urban watershed, concluding that tolerance is strain-specific and not predictable by taxonomic affiliation at the genus level. Orji et al. (2021) observed similar patterns in halotolerant and metallotolerant bacteria isolated from saline environments, where strains of the same genus exhibited markedly different bioremediation capabilities.

The functional difference between JSRA_S05 and JSRA_N14 could be explained by the differential acquisition of Cd tolerance genes present in mobile genetic elements undetectable by 16S rRNA or BOX-PCR, such as efflux operons, metallothioneins, or biotransformation systems (Bravo and Braissant, 2022). JSRA_S05, isolated from soil with direct exposure to Cd, would have been subjected to greater selective pressure to acquire these elements, whereas JSRA_N14, in the protected nodular niche, may not have developed the same adaptive capacity.

### 4.6 Association with the host: *Quararibea* sp. as a reservoir of tolerant strains

The two strains associated with *Quararibea* sp.—JSRA_S08 (soil, highly tolerant, hormesis) and JSRA_R15 (root, tolerant, IC₅₀ = 301.7 ppm)—were consistently among the most tolerant isolates. Castillo et al. (2016) documented that *Quararibea* sp. accumulates heavy metals in its tissues, implying that its rhizosphere and root tissues are exposed to elevated metal concentrations, creating a selective microenvironment for tolerant bacteria. Chi et al. (2024) demonstrated that growth-promoting endophytes modulate soil ecological characteristics during Cd phytoremediation, reinforcing the hypothesis of synergistic plant-microorganism interaction in contaminated environments.

JSRA_R15 is particularly noteworthy: the only strain isolated from the root endosphere of a non-leguminous species, identified as *Agrobacterium leguminum* (Rhizobiaceae). Although *Agrobacterium* belongs to the Rhizobiaceae family, it is not a nitrogen-fixing rhizobium, which is consistent with the central finding of the absence of rhizobia sensu stricto. Its IC₅₀ of 301.7 ppm positions it as a priority candidate for plant-assisted bioremediation.

### 4.7 Implications for bioremediation and future prospects

The three candidate strains identified for future functional evaluation—JSRA_S05 (*K. variicola*), JSRA_S08 (*Glutamicibacter* sp.) and JSRA_R15 (*A. leguminum*)—combine high *in vitro* physiological tolerance with taxonomic and source diversity. While in vitro tolerance alone does not establish bioremediation potential, native strains may, in principle, offer adaptive advantages over non-native ones in terms of survival and competitiveness in the local soil (Bravo and Braissant, 2022; Feria-Cáceres et al., 2022; Shen et al., 2023). However, prior to any bioinoculant application, these strains require validation through assays of biosorption and bioaccumulation, metal speciation analyses, plant inoculation experiments and soil microcosm or greenhouse trials.

However, the study’s limitations must be acknowledged. Tolerance assessment was conducted in vitro in TY medium, conditions that differ from the edaphic environment where Cd interacts with the soil matrix. The TI and IC₅₀ values reflect intrinsic physiological tolerance under controlled conditions, not necessarily field performance. Furthermore, the sample size (*n* = 12) limits the generalizability of the patterns, particularly the association between origin and tolerance. The culture-based approach may have underestimated the actual diversity, particularly of slow-growing rhizobia.

Future studies should focus on: (i) genomic characterization of candidate strains to identify molecular determinants of Cd tolerance; (ii) evaluation of biosorption and bioaccumulation capacities; (iii) greenhouse inoculation trials with *T. cacao* and *Inga* sp. in contaminated soils; (iv) complementing the study with metagenomic sequencing to assess the actual proportion of rhizobia versus NREs in nodules under Cd stress; and (v) expanding sampling to other cocoa plots in the Peruvian Amazon.

### 4.8 Limitations of the study

Several limitations of the present study should be made explicit. First, the entire dataset is culture-dependent, recovered on YMA medium under aerobic conditions; this approach is known to under-represent slow-growing rhizobia, particularly bradyrhizobia, which typically dominate nodules of tropical leguminous trees. The absence of cultivable rhizobia sensu stricto reported here cannot therefore be equated with community-level absence and requires confirmation by culture-independent approaches (e.g., 16S rRNA amplicon sequencing or metagenomics from nodule tissue, or qPCR of symbiotic markers such as *nodA* and *nifH*). Second, the final set of 12 strains is the product of a sequential selection process (93 cultivable isolates → 53 Cd-tolerant → 33 consistently tolerant across all five concentrations → 23 sequenced under budget constraints → 12 with high-quality 16S rRNA reads); the isolates analyzed are therefore doubly enriched in cultivable, Cd-tolerant phenotypes and are not representative of the underlying bacterial community. Diversity indices (Shannon, Simpson) reach their maximum theoretical values as a mathematical consequence of the 12 unique genotypes recovered, and should not be interpreted as community-level diversity. Third, the field identity of the sampled structures as authentic *Inga* sp. root nodules was based on macroscopic morphological criteria following Somasegaran and Hoben (1994); no histological sectioning, internal-pigmentation analysis or *nifH*/leghaemoglobin verification was carried out, and we therefore cannot strictly exclude the possibility that a subset of the sampled structures corresponded to other root-associated swellings. Fourth, taxonomic identification was based on a single near-full-length 16S rRNA marker (universal primers fD1/rD1; budget did not allow MLSA or whole-genome sequencing); species-level names reported here should be read as the closest type-strain match in the 16S database and not as definitive species delimitation, especially for *Klebsiella*, *Pseudomonas*, *Achromobacter*, *Cedecea,* and *Glutamicibacter*. Fifth, *in vitro* Cd tolerance was assessed by OD₆₀₀ in TY broth using uninoculated medium with Cd as the blank for each concentration; we did not perform parallel CFU counts to verify the correlation between OD₆₀₀ and viable cell number under metal stress, did not measure medium pH after Cd addition, and did not specifically control for Cd-induced precipitation or cell aggregation. These factors may bias growth-inhibition estimates and should be addressed in follow-up assays. Sixth, in vitro Cd tolerance demonstrates the physiological capacity to grow in the presence of the metal under controlled conditions; it does not by itself establish metal sequestration, immobilization, transformation, reduced plant uptake or field efficacy, and the strains identified here should be regarded as candidates for future functional evaluation, not as validated bioinoculants. Seventh, soil Cd and physicochemical properties of the study area (Table 1) come from a previous characterization of the same agroforestry zone under the FITOCADMIO project; the 36 plots sampled in the present study were not individually re-analyzed for soil Cd, so site-by-site associations between local Cd levels and bacterial tolerance phenotypes cannot be drawn here. Finally, sampling was carried out within a single province (Bagua) and a limited number of cocoa agroforestry farms; broader spatial replication will be required before any of these findings can be generalized to other regions of the Peruvian Amazon.

## 5 Conclusion

The cultivable bacteria recovered from *Inga* sp. nodules, *Quararibea* sp. roots and rhizosphere soil in cocoa agroforestry systems in Bagua, Amazonas, span three bacterial phyla (Proteobacteria, Firmicutes, Actinobacteria) and five classes, with 12 unique BOX-PCR genotypes. As each isolate represents a single genotype, the calculated Shannon and Simpson indices reflect the maximum theoretical diversity for this isolate set rather than community-level diversity, and should be interpreted as descriptive of the cultivable fraction only.

The absence of cultivable rhizobia sensu stricto and the dominance of non-rhizobial endophytes among the isolates recovered from *Inga* sp. nodules—members of *Achromobacter*, *Pseudomonas*, *Klebsiella*, *Priestia*, and *Glutamicibacter*, adds to growing evidence that legume nodules can host complex, non-rhizobial bacterial communities. Whether classical rhizobia are truly absent or simply not retrieved under the cultivation conditions employed cannot be resolved with the present dataset and should be assessed in future work using culture-independent approaches and symbiotic-marker analyses.

The isolates exhibit contrasting cadmium response phenotypes, ranging from high sensitivity (TI = 0.190) to high tolerance with a clear hormetic response in JSRA_S05 (TI = 1.235 at 25 ppm Cd) and a possible hormetic tendency in JSRA_S08 (TI = 1.012 at 50 ppm Cd). The discordance between classifications based on TI and IC₅₀ (Spearman *ρ* < 0.1) demonstrates that both parameters capture complementary dimensions of tolerance and underscores the need for multidimensional evaluation systems.

Cadmium tolerance is strain-specific and not predictable by taxonomic affiliation, as evidenced by the opposite response of two *K. variicola* strains (highly tolerant JSRA_S05 vs. highly sensitive JSRA_N14), suggesting that accessory genomic factors determine adaptive capacity to metal stress.

The strains JSRA_S05 (*K. variicola*), JSRA_S08 (*Glutamicibacter* sp.), and JSRA_R15 (*A. leguminum*), which combine high in vitro Cd tolerance with taxonomic and source diversity, are proposed as candidates for future functional evaluation (biosorption, bioaccumulation, plant inoculation and soil microcosm or greenhouse trials), which will be required before any bioinoculant application can be considered.

## Data Availability

The datasets presented in this study can be found in online repositories. The names of the repository/repositories and accession number(s) can be found in the article/Supplementary material.
