# Supplementary material for: Cadmium tolerance and taxonomic diversity of cultivable bacteria isolated from Inga sp. nodules, Quararibea sp. roots and rhizosphere soil in cocoa agroforestry systems of the Peruvian Amazon
Source: Front Microbiol. 2026 Aug 7;17:1845000. doi: 10.3389/fmicb.2026.1845000 (PMC13494932; doi:10.3389/fmicb.2026.1845000)
Supplement: Supplementary file 1 [file Table_1.docx]

Supplementary Material

# 1 Supplementary Tables

**Supplementary Table S1.** Plot-level metadata of the 36 sampling units established in cocoa agroforestry systems of Bagua province, Amazonas, Peru. Sample codes follow the format JSRA[plot number]. The strain codes used in the main text (e.g. JSRA_N05, JSRA_R15, JSRA_S08) include a compartment prefix (N = nodule, R = root, S = rhizosphere soil) and the plot number from which the cultivable, Cd-tolerant, high-quality-sequence isolate was recovered. The present study focused on isolates obtained from Inga sp. (Guaba) and Quararibea sp. (Zapote); plots with other tree species are reported here for completeness of the sampling effort. Soil Cd concentrations were not measured plot-by-plot in this study; the edaphic context of the area is summarised in Table 1 of the main text. UTM coordinates are given in zone 17M (WGS84 datum).

| **Plot** | **Sample code** | **District – Locality** | **Altitude (m)** | **UTM coordinates (zone 17M)** | **Sample type** | **Host tree species (common name; scientific name)** |
| --- | --- | --- | --- | --- | --- | --- |
| 1 | JSRA01 | Imaza – Chiriaco | 289.8 | 798659 / 9431753 | Soil | Pajuro |
| 2 | JSRA02 | Imaza – Chiriaco | 289.8 | 798661 / 9431754 | Soil | Cacao Criollo |
| 3 | JSRA03 | Imaza – Chiriaco | 289.8 | 798662 / 9431754 | Soil | Cacao |
| 4 | JSRA04 | Imaza | 287.6 | 798309 / 9430539 | Soil + nodule | Guaba (*Inga* sp.) |
| 5 | JSRA05 | Imaza | 288.6 | 798310 / 9430539 | Soil + nodule | Guaba (*Inga* sp.) |
| 6 | JSRA06 | Imaza | 287.6 | 798308 / 9430540 | Soil + nodule | Guaba (*Inga* sp.) |
| 7 | JSRA07 | Imaza | 290.2 | 798308 / 9430540 | Soil + nodule | Guaba (*Inga* sp.) |
| 8 | JSRA08 | Imaza – Chiriaco | 289.8 | 798659 / 9431754 | Soil + root | Zapote (*Quararibea* sp.) |
| 9 | JSRA09 | Imaza – Chiriaco | 289.8 | 798659 / 9431754 | Soil + root | Zapote (*Quararibea* sp.) |
| 10 | JSRA10 | Copallín – Cajaruro | 946.8 | 786919 / 9371915 | Soil + nodule | Guaba (*Inga* sp.) |
| 11 | JSRA11 | Copallín – Cajaruro | 946.8 | 786919 / 9371922 | Soil + nodule | Guaba (*Inga* sp.) |
| 12 | JSRA12 | Copallín – Cajaruro | 946.8 | 786919 / 9371920 | Soil + nodule | Guaba (*Inga* sp.) |
| 13 | JSRA13 | Copallín – Cajaruro | 292.8 | 787074 / 9370891 | Soil + nodule | Guaba (*Inga* sp.) |
| 14 | JSRA14 | Copallín | 873.9 | 786685 / 9373192 | Soil + nodule | Guaba (*Inga* sp.) |
| 15 | JSRA15 | Copallín | 900.4 | 786738 / 9373104 | Soil + root | Zapote (*Quararibea* sp.) |
| 16 | JSRA16 | Copallín | 893.8 | 786731 / 9373096 | Soil + nodule | Guaba (*Inga* sp.) |
| 17 | JSRA17 | Copallín | 882.4 | 786701 / 9373093 | Soil + nodule | Guaba (*Inga* sp.) |
| 18 | JSRA18 | Copallín | 868.3 | 786797 / 9373290 | Soil + root | Zapote (*Quararibea* sp.) |
| 19 | JSRA19 | Copallín | 869.3 | 786797 / 9373291 | Soil + nodule | Guaba (*Inga* sp.) |
| 20 | JSRA20 | Copallín | 873.8 | 786930 / 9373332 | Soil + nodule | Guaba (*Inga* sp.) |
| 21 | JSRA21 | Copallín | 893.1 | 786933 / 9373336 | Soil + nodule | Guaba (*Inga* sp.) |
| 22 | JSRA22 | Copallín | 965.4 | 787011 / 9371997 | Soil + nodule | Guaba (*Inga* sp.) |
| 23 | JSRA23 | Copallín | 962.0 | 787001 / 9371988 | Soil + nodule | Guaba (*Inga* sp.) |
| 24 | JSRA24 | Copallín | 965.4 | 787000 / 9371988 | Soil + nodule | Guaba (*Inga* sp.) |
| 25 | JSRA25 | Copallín | 965.4 | 787000 / 9371988 | Soil + nodule | Guaba (*Inga* sp.) |
| 26 | JSRA26 | Copallín – La Lluhuana | 923.0 | 786996 / 9372001 | Soil + nodule | Guaba (*Inga* sp.) |
| 27 | JSRA27 | Copallín – La Lluhuana | 950.6 | 787034 / 9372000 | Soil + nodule | Guaba (*Inga* sp.) |
| 28 | JSRA28 | Copallín – Cajaruro – La Lluhuana | 973.4 | 787026 / 9372057 | Soil + nodule | Guaba (*Inga* sp.) |
| 29 | JSRA29 | Copallín – Cajaruro – La Lluhuana | 973.4 | 787026 / 9372057 | Soil + nodule | Guaba (*Inga* sp.) |
| 30 | JSRA30 | Copallín – La Lluhuana | 963.1 | 787018 / 9372087 | Soil + nodule | Guaba (*Inga* sp.) |
| 31 | JSRA31 | Copallín – Cajaruro | 962.3 | 786990 / 9372081 | Soil + nodule | Guaba (*Inga* sp.) |
| 32 | JSRA32 | Copallín – Cajaruro | 965.1 | 786994 / 9372065 | Soil + nodule | Guaba (*Inga* sp.) |
| 33 | JSRA33 | Copallín – Cajaruro | 957.4 | 786999 / 9372048 | Soil + nodule | Guaba (*Inga* sp.) |
| 34 | JSRA34 | Copallín – Cajaruro | 957.4 | 786999 / 9372048 | Soil + nodule | Guaba (*Inga* sp.) |
| 35 | JSRA35 | Copallín – Cajaruro | 962.1 | 786999 / 9372044 | Soil + nodule | Guaba (*Inga* sp.) |
| 36 | JSRA36 | Copallín – Cajaruro | 945.9 | 787011 / 9372020 | Soil + nodule | Guaba (*Inga* sp.) |

Sample type indicates which compartments were processed per plot: "Soil" = rhizosphere soil only; "Soil + nodule" = rhizosphere soil and root nodules; "Soil + root" = rhizosphere soil and root tissue (endosphere). Plot-level codes (JSRA01–JSRA36) refer to sampling units. Strain-level codes used in the main text combine a compartment letter (N, R or S) with the corresponding plot number — for example, JSRA_N05 denotes the cultivable, Cd-tolerant, high-quality-sequence isolate obtained from the nodule compartment of plot 5; JSRA_S05 denotes the corresponding soil isolate from the same plot; JSRA_R15 denotes the root-endosphere isolate from plot 15. Combinations not listed in the final 12-strain set (e.g. there is no JSRA_R05 or JSRA_N04 in the dataset) reflect compartments that either did not yield Cd-tolerant cultivable bacteria or yielded isolates whose 16S rRNA sequences did not pass the quality threshold (see Methods 2.3, "Strain selection workflow").

# 2 Supplementary Figure S1

**Supplementary Figure S1.** Detailed dose-response profile of the two isolates showing Tolerance Index (TI) values above 1.0 at one or more cadmium concentrations: JSRA_S05 (Klebsiella sp., closest 16S match K. variicola) and JSRA_S08 (Glutamicibacter sp.). The red dashed horizontal line indicates the hormesis threshold (TI = 1.0). Triangles mark TI values > 1.0; circles mark TI < 1.0. Error bars represent standard error of the mean across three biological replicates × two measurement times (24 and 48 h). JSRA_S05 shows a clear biphasic hormetic profile with TI = 1.235 at 25 ppm and TI = 1.178 at 50 ppm, followed by progressive inhibition at higher concentrations. JSRA_S08 shows only a borderline value at 50 ppm (TI = 1.012, i.e. 1.2% above control) that falls within typical OD600 assay variability; it is therefore interpreted as a possible hormetic tendency rather than confirmed hormesis (see Methods 2.7 and Discussion 4.4).
